# Effects of human concurrent aerobic and resistance training on cognitive health: A systematic review with meta-analysis

**DOI:** 10.1016/j.ijchp.2025.100559

**Published:** 2025-03-20

**Authors:** Mingyang Zhang, Wangfan Fang, Jiahong Wang

**Affiliations:** a School of Sport Science, Jishou University, Jishou, China; b Soochow University Think Tank, Soochow University, Suzhou, China

**Keywords:** Exercise, Concurrent training, Cognition, Mild cognitive impairment, Older adults, Health

## Abstract

**Background:**

The rising prevalence of cognitive decline and neurodegenerative diseases, projected to affect 150 million individuals by 2050, highlights the urgent need to enhance neurocognitive health. While both aerobic and resistance training are recognized as effective strategies, their combined effects on cognition remain underexplored.

**Objective:**

This study aimed to determine if concurrent aerobic and resistance training (CT) is effective in enhancing cognitive function.

**Methods:**

Seven English and three Chinese databases were searched from inception to August 2024. Randomised controlled trials (RCTs) examining the effects of CT on global cognition across diverse populations were included. A meta-analysis was performed using a random-effects model in R and Stata, supplemented by subgroup and meta-regression analyses to explore variability.

**Results:**

The meta-analysis included 35 RCTs with 5,734 participants, revealing a positive effect of CT on global cognition (g = 0.32, 95% CI: 0.17–0.46, p < 0.001). Notably, older adults (≥65 years) exhibited greater cognitive benefits (g = 0.33; 95% CI: 0.14–0.51, p < 0.05) compared to younger populations. Significant effects were also observed in clinical populations (g = 0.28; 95% CI: 0.11–0.46, p < 0.001). Exercise frequency and duration positively influenced outcomes, with medium-length interventions (13–26 weeks) demonstrating significant effects (g = 0.21; 95% CI: 0.05–0.37, p = 0.011).

**Conclusion:**

The findings indicate that CT significantly enhances cognitive health, particularly in older adults and clinical populations. Prioritizing strength training, implementing short- to medium-term interventions (4–26 weeks), and maintaining session durations of 30–60 minutes are crucial for optimizing cognitive benefits.

## Introduction

Cognitive function—spanning memory, attention, executive function, and processing speed—is essential across the lifespan, influencing school readiness (Blair, 2002; Diamond & Lee, 2011), academic achievement (Rohde & Thompson, 2007; Welsh et al., 2010), job performance (Lang et al., 2010), and overall health in later life (Ludyga et al., 2020). However, aging and neurodegenerative diseases (e.g., Alzheimer's disease) threaten neurocognitive health, defined as the optimal state of these cognitive domains that enable individuals to maintain independence and quality of life (Stillman et al., 2020). Indeed, healthy aging encompasses not only the absence of disease or disability but also the ability to maintain functional capabilities in older age (Beard et al., 2016; Organization, 2015; Rowe & Kahn, 1997). Given the dynamic and nonlinear nature of cognitive trajectories throughout the lifespan (Turrini et al., 2023), it is crucial to differentiate between synergistic adaptations and natural cognitive growth when evaluating the benefits (Robinson et al., 2023). Critically, cognitive health extends beyond mere enhancement; it encompasses both the improvement of current capacities and the delay of age- or pathology-related decline (Deckers et al., 2024; Kaliman et al., 2011; Stern, 2002). This dual perspective aligns with the World Health Organization's holistic definition: “the dynamic maintenance of cognitive resilience to support well-being and societal engagement across the lifespan (Organization, 2021)”. Moreover, mental disorders, such as depression and schizophrenia, are increasingly recognized as risk factors for accelerated cognitive decline (Kandola et al., 2019). These conditions are associated with dysregulated neurotrophic factors, chronic inflammation, and impaired neurogenesis, which may exacerbate cognitive deficits (Pereira et al., 2007). However, exercise-induced brain-derived neurotrophic factor (BDNF) elevation (Kim et al., 2019) and hypothalamic-pituitary-adrenal (HPA) axis modulation (Schuch, Vancampfort, Sui, et al., 2016) may concurrently alleviate depressive symptoms and enhance memory consolidation—a hypothesis supported by trials integrating physical exercise with cognitive-behavioral therapy (Biazus-Sehn et al., 2020; Nuechterlein et al., 2018). Understanding the interplay between mental health and exercise-induced cognitive benefits is critical for optimising programs. Therefore, the current question of how to achieve successful aging is attracting the attention of accumulating healthcare providers and policymakers (Jia et al., 2020).

Lifelong engagement in physical activity has emerged as a promising strategy to delay (Paillard, 2015; Zimmerman et al., 2014), prevent (Sujkowski et al., 2022; Turrini et al., 2023), or even reverse (Kaliman et al., 2011; Xu et al., 2021) cognitive decline (Stillman et al., 2020). Conversely, sedentary behavior was seen as a major threat for physical and cognitive health (Piercy et al., 2018; Zou et al., 2024). Exercise, characterized by its accessibility and minimal side effects, offers benefits that beyond mere physical fitness (Larson & Bruce, 1987; Penedo & Dahn, 2005). Recent research highlights the growing interest in exercise programs aimed at improving cognitive health (A'Naja et al., 2024; Ren et al., 2024). Among the various types of exercise, aerobic/endurance training (AET) and strength/resistance training (SRT) have demonstrated significant benefits for psychophysiological health (Al-Mhanna et al., 2024; Al-Mhanna et al., 2023; Batrakoulis & Fatouros, 2022), particularly in older adults and clinical populations (Ballesteros et al., 2024; Chow et al., 2021; Erickson et al., 2019; Gallardo-Gómez et al., 2022). AET has been shown to enhance cardiovascular health and cognitive function, reducing risks related to brain health and improving executive control (Al-Mhanna et al., 2023; Batrakoulis et al., 2022). On the other hand, SRT contributes to improved muscle function and supports brain adaptability (McArdle et al., 2010; Nagamatsu et al., 2012). Furthermore, concurrent training (CT), which combines aerobic and resistance training, has emerged as a promising approach due to its potential for delivering combined benefits (Al-Mhanna et al., 2024). Both AET and SRT are characterized by automatic repetition, with higher metabolic energy and lower cognitive engagement (Ludyga et al., 2022; Netz, 2019; Tomporowski & Pesce, 2019; Voelcker-Rehage & Niemann, 2013). Recent studies (Meijer et al., 2020) emphasizing the impact of cognitively demanding exercise that involve mental involvement (such as mind-body exercises and skill training) might introduce confounding factors in the cognitive outcome. In contrast, exploring the ‘black box’ of how energy demand-dominant exercise improve cognition is far more intriguing.

A substantial body of evidence-based research investigates the effects (Cammisuli et al., 2017; Ren et al., 2023) and mechanisms (Liang et al., 2021; Tari et al., 2019) by which both AET (Cammisuli et al., 2017; Stern et al., 2019) and SRT (Coelho-Junior et al., 2022; Mavros et al., 2017) enhance cognitive function. The simplicity of AET has led to its more widespread use in research settings for enhancing cognitive health compared to SRT (Ciria et al., 2023; de Asteasu et al., 2017; Song et al., 2018). Most large-scale meta-analyses examining the relationship between exercise training and cognitive function (Chen et al., 2020; Haverkamp et al., 2020; Northey et al., 2018; Zhang et al., 2023) have included more studies on AET than on SRT. Specifically, both AET and SRT improve global cognition (Han et al., 2023), attention (Dunsky et al., 2017; Hacker et al., 2020), memory (Babaei et al., 2013; Cassilhas et al., 2012; Erickson et al., 2011; Makino et al., 2021), information processing speed (Eckardt et al., 2020; Sandroff et al., 2015), cognitive flexibility (Netz et al., 2007; Segabinazi et al., 2020), and executive functions, including working memory (Ludyga et al., 2022; Pontifex et al., 2009), planning (Y. K. Chang, P. W. Ku, et al., 2012; Y. K. Chang, C. Y. Pan, et al., 2012; Hung et al., 2013), and inhibitory control (Amatriain‐Fernández et al., 2021; Ludyga et al., 2018; Wilke et al., 2020). The acute and chronic effects of AET benefit cognitive health across various age groups, including children and adolescents (Davis et al., 2007; Harveson et al., 2016; Robinson et al., 2023), young and middle-aged adults (Dunsky et al., 2017; Stern et al., 2019; Stroth et al., 2009), and older adults (Best et al., 2015; Garcia-Soto et al., 2013), as well as healthy (Nouchi & Kawashima, 2014) and diverse clinical populations (Cerrillo‐Urbina et al., 2015; McDonnell et al., 2011; Papatsimpas et al., 2023; Zhang et al., 2022). Conversely, both acute and chronic studies indicate that SRT improves cognitive function across age groups; however, the effects of exercise on attention improvement and acute responses in adolescents are not fully understood. Furthermore, inconsistencies exist in findings related to the global cognitive benefits of AET (Young et al., 2015), improvements in global cognition (J. R. Ruiz et al., 2015), memory (Cavalcante et al., 2020), and inhibition (Chang & Etnier, 2009) associated with SRT, as well as the effects on working memory from both exercise modalities (Landrigan et al., 2020; Zhidong et al., 2021). Scientific evidence on the relationship between exercise and cognition has steadily increased over the past half-century. Current research explores the dose-response relationship (Engeroff et al., 2019; McCartney et al., 2021) and optimal exercise prescriptions (Huang et al., 2022) in depth. However, existing literature primarily emphasizes the individual effects of these exercise modalities, overlooking potential synergistic or interference effects from their combined application.

The concept of “interference effects” in CT has been extensively discussed at the physiological level (Coffey & Hawley, 2017; Fyfe et al., 2014; Huiberts et al., 2024; Lundberg et al., 2022; Wadsworth et al., 2022). However, its implications for neurocognitive adaptation have received limited attention (Hickson, 1980). Early research suggests that exercise induces specific adaptations in cognitive functions (Guadagni et al., 2020; Netz, 2019; Smiley-Oyen et al., 2008; Tsai et al., 2019; Voelcker-Rehage & Niemann, 2013), while recent systematic reviews indicate that exercise-related cognitive benefits may be more extensive than previously understood (Ludyga et al., 2020). AET enhances brain health by promoting the growth of new neurons in the hippocampus and blood vessel formation in the cortex, driven by increased levels of brain-derived neurotrophic factor (BDNF) (Tsai et al., 2014) and vascular endothelial growth factor (VEGF) (Guan & Yan, 2022; Voss et al., 2013). In contrast, SRT improves neuromuscular efficiency and activates the prefrontal cortex through mechanisms involving protein synthesis and the release of muscle-derived factors like irisin (Coelho-Junior et al., 2022; Peng et al., 2017). Additionally, AET enhances cerebral blood flow and glucose metabolism, supporting executive functions (Guadagni et al., 2020), while SRT increases levels of cathepsin B, a muscle-derived factor that reduces brain inflammation (Kim et al., 2019). At the macro-mechanism, both AET and SRT induce adaptive changes in brain structure and function, such as increased gray matter volume, improved white matter integrity, and enhanced connectivity between brain regions, all of which contribute to better cognitive performance (Coelho-Junior et al., 2022; Lopez-Ortiz et al., 2021; Peng et al., 2017; Silva et al., 2024).

Although acute CT may lead to competition for metabolic resources, long-term integration of these training modalities likely amplifies neuroprotection by targeting multiple systems—structural (gray matter), functional (brain connectivity), and molecular (BDNF and irisin) (Coffey & Hawley, 2017). This multifaceted approach aligns with the “cognitive reserve” model, which posits that diverse stimuli build resilience against age-related cognitive decline (Stern, 2002). However, potential interference effects between AET and SRT warrant attention. For example, animal studies suggest that SRT, depending on intensity, may reduce some cognitive benefits and neurogenesis triggered by AET (Lan et al., 2018). The distinct biological mechanisms or inflammatory responses associated with SRT might counteract certain cognitive gains from AET. Thus, understanding the balance between synergy and interference in CT is crucial for designing exercise programs that maximise cognitive benefits, both for elite athletes and broader populations in public health.

In this study, we conducted a moderation analysis of the characteristics of CT rather than according to the FITT-VP principles (Zhang et al., 2023). Consequently, the systematic review and meta-analysis included a wide range of studies without restrictions on publication date or age. The study primarily examined four key issues: (1) the impact of CT on overall cognitive function, reflecting cognitive health across the lifespan; (2) the influence of participant characteristics, including variations across different age groups and health statuses; (3) the moderating effects of training variables, such as intervention duration, frequency, session length, exercise order, and CT configurations, to aid in formulating appropriate training prescriptions; and (4) the impact of study design, considering the nature of the control group and differences in cognitive tasks.

## Methods

This systematic review and meta-analysis adhered to the Preferred Reporting Items for Systematic Reviews and Meta-Analysis (PRISMA) guidelines to ensure methodological transparency and rigorous reporting of results (Liberati et al., 2009). The protocol for this study was pre-registered at PROSPERO (ID: CRD42024593636).

### Literature search

Seven English databases (PubMed, Web of Science, EMBASE, CINAHL, Scopus, Google Scholar, Cochrane Library) and three Chinese databases (China National Knowledge Infrastructure, Baidu Scholar and WanFang Data) were independently searched from inception to January 2024, and an updated search was performed on August 2024. Additionally, we conducted a search through Google Scholar to identify unpublished literature and papers not indexed in the Science Citation Index. The search employed a strategic combination of Medical Subject Headings (MeSH) and free-text keywords pertinent to cognition, strength / resistance training, aerobic exercises. The details of search strategy are available in **Supplementary file S1**. To enhance the comprehensiveness of strategy, the reference lists of identified studies were manually reviewed, and citation tracking was conducted using Elicit (Whitfield & Hofmann, 2023).

### Study selection

Two independent reviewers (ZMY and FWF) performed a multi-step screening of titles, abstracts, and full texts. Discrepancies were resolved through discussion, with a third reviewer consulted if needed. Only studies deemed relevant by both reviewers advanced to full-text review.

### Eligibility criteria

The inclusion and exclusion criteria were assessed using the PICOS framework (Participants, Intervention, Comparators, Outcomes, Study Design) (Liberati et al., 2009). Eligibility criteria were detailed in Table 1. To investigate the interference effects of CT, as observed in animal models, this review encompasses a diverse range of cognitive statuses and age to address the limited number of published randomised controlled trials (RCTs). By incorporating a variety of populations and study designs, this review aims to provide the first comprehensive evaluation of the effects of CT on cognitive health in human.

**Table 1:** Eligibility criteria for meta-analysis.

Category	Inclusion criteria	Exclusion criteria
Participants	Human with cognitively impaired and unimpaired. There were no restrictions on baseline cognitive status, allowing for a broad inclusion of participants with varying cognitive abilities	Animal model were excluded from meta-analysis
Intervention	Combined aerobic and strength training (AET + SRT) programs. Programs needed to be explicitly stated as fully supervised and lasting at least 4 weeks to ensure the isolated effects of exercise could be measured.	Single-mode exercise training interventions
Comparators	Both active and passive control group	Absence of a control group, or involving medical treatments and placebo
Outcomes	Global cognitive function and cognitive impairment: Mini-Mental State Examination (MMSE), Modified Mini-Mental State Exam (3MSE), Montreal Cognitive Assessment (MoCA), Addenbrooke's Cognitive Examination Revised (ACE-R), Paced Auditory Serial Addition Task (PASAT), and MATRICS Consensus Cognitive Battery (MCCB), and Severe Impairment Battery – Short Form (SIB-S), Alzheimer's Disease Assessment Scale – Cognitive Sub-Scale (ADAS-Cog)	Lack of baseline and/or post-intervention data
Study design	peer-reviewed publication, randomised controlled trials	Non-randomised controlled trials, conference abstracts, clinical protocols, observational or quasi-experimental studies

Note: AET, aerobic / endurance / cardiovascular / cardiorespiratory exercise training; SRT, strength / resistance exercise training; AD, Alzheimer's disease.

### Quality assessment

To assess the certainty of evidence for key outcomes, we applied the Grading of Recommendations Assessment, Development and Evaluation (GRADE) guidelines (Group, 2004). The GRADE approach evaluates evidence quality based on five domains: risk of bias, inconsistency, indirectness, imprecision, and publication bias. Furthermore, the methodological rigor of the studies included in this analysis was rigorously evaluated using the Cochrane Risk of Bias Tool (robvis), a standardised instrument designed to assess critical methodological aspects, including sequence generation, allocation concealment, participant and personnel blinding, outcome assessor blinding, and data completeness (McGuinness & Higgins, 2020). Each study was systematically categorised according to its risk of bias as low, unclear, or high for each criterion. Discrepancies in the risk of bias assessments between the two reviewers were resolved independently through a consensus process; if necessary, a third reviewer was consulted to ensure objectivity.

### Data extraction and synthesis

Data extraction was conducted utilizing a standardised form to systematically collect various characteristics, including bibliographic details (first author and year of publication), intervention variables (i.e., frequency, sessional and weekly duration, intensity, volume, length, same session and additional training between AET and SRT), participant characteristics (i.e., sample size, sex, age, healthy status), control conditions (i.e., health education, and routine care/treatment), outcomes [the statistics at the endpoint of the intervention for estimating effect sizes (ES)], study design, and main findings. Based on prior literature (Norton et al., 2010) and guidelines (Baechle & Earle, 2008; Ratamess, 2021), the exercise intensities of AET and SRT were standardised and categorised as sedentary (S), light (L), moderate (M), vigorous (V), and high (H).

Means and standard deviations (SDs) for cognitive measures at baseline and follow-up were extracted for both intervention and control groups. In instances of incomplete reporting of pertinent statistical data, calculations and synthesis were performed using standard errors, within-group confidence intervals (CI), medians, ranges, and p-values, adhering to the formulas delineated in the Cochrane Handbook (Higgins & Green, 2008). Furthermore, missing or improperly formatted data were obtained by contacting the corresponding authors. If means and SDs for each group were not reported, the authors of the primary studies were approached to request baseline and post-intervention data. When data were presented graphically and no additional information was provided upon request, the data were extracted using GetData Graph Digitizer version 2.26 (Sydney, Australia) (Sun et al., 2020).

In cases where multiple articles derived from the same study reported identical or overlapping outcomes, all were included in the systematic review. Additionally, if a study conducted cognitive assessments both post-intervention and during follow-up, only the post-intervention data were extracted. Given that lower ADAS-Cog scores indicate improved cognitive health, the data were inverted to ensure that higher transformed scores consistently reflected enhanced cognitive function across all included measures.

### Data coding

CT prescriptions were coded as categorical variables according to established guidelines (García-Hermoso et al., 2018; Northey et al., 2018; Zhang et al., 2023). The classifications for the CT are detailed in **Supplementary file S2**. The characteristics of CT included training configuration (*strength training, aerobic exercise, circle-based,* and *separation*), additional training (beyond SRT and AET: *yes* or *no*), concurrent design (*same week* or *same session*), session duration (*short*: 30–45 mins, *medium*: 60 mins, *long*: 90 mins, or *unclear*), intervention length (*short*: 4–12 weeks, *medium*: 13–26 weeks, *long*: >26 weeks). Additionally, the frequency of training sessions per week was categorised as *low* (1–2), *medium* (3–4), or *high* (≥5) for both groups.

Global cognition, an umbrella term that encompasses various cognitive domains, including memory, attention, executive function, and visuospatial abilities (Huang et al., 2022; Zhang et al., 2023), was assessed using a range of neuropsychological tests (*MMSE, 3MSE, ADAS-Cog, MoCA*), categorised according to cognitive moderators.

Regarding age moderators, participant age was classified into two categories based on the reports from each included study: *young and middle adulthood* (18–65 years) and *older adults* (>65 years).

Finally, recognizing that the characteristics of the control group may influence the effects of exercise interventions on cognitive health, the control conditions were classified into six categories based on the included studies: *health education, no exercise, other exercises, routine activity, routine treatment / care*, and *social activity* (Northey et al., 2018).

### Statistical analysis

Statistical analysis was conducted with Stata version 18 and R version 4.2.2 software, employing the metafor package (Viechtbauer, 2010). Sandardised mean differences (SMDs) were calculated based on the MD from baseline to follow-up and pooled standard deviations. An inverse variance-weighted random-effects model was applied to the overall ES and 95% CI. A positive ES indicated a greater cognitive benefit in the exercise group compared to the control group, while a negative ES suggested superior cognitive improvement in the control group. Heterogeneity (τ²) was estimated using the restricted maximum likelihood estimator (REML) (Viechtbauer, 2005). To further evaluate heterogeneity, the Q test (Cochran, 1954) and the I² statistic (Higgins & Thompson, 2002) were calculated, with I² values of 20%, 50%, and 75% indicating low, moderate, and high heterogeneity, respectively (Higgins & Green, 2008). If any degree of heterogeneity is detected (i.e., τ² > 0, irrespective of the Q-test results), a prediction interval for the true outcomes is also provided. Studentized residuals and Cook's distances were utilized to assess whether any studies were outliers or influential within the context of the model.

Meta-regression analyses were conducted to investigate potential sources of heterogeneity. Each moderator was incorporated into a random-effects univariate meta-regression analysis utilizing maximum likelihood estimation (Hedges & Olkin, 2014). Subgroup analyses were performed based on single-group sample size (n<50 vs. n≥50) and health status of participants (healthy populations vs. clinical populations), which were conducted to examine potential effect modifiers, including sex, age, intervention characteristics, duration, and frequency. Random-effects models utilizing REML were employed to calculate the ΔES and 95% CI for moderator variables (Gordon et al., 2018). Funnel plots were visually inspected for asymmetry (Duval & Tweedie, 2000). Additionally, Begg rank correlation tests and Egger's test values, along with 95% CI, were calculated using the standard error of the observed outcomes as predictors to statistically assess publication bias (Begg & Mazumdar, 1994; Egger et al., 1997). Small-sample bias was deemed present when the funnel plot exhibited asymmetry and the intercept of Egger's test was significantly different from zero (p < 0.10).

## Results

### Study identification

**Fig. 1:**
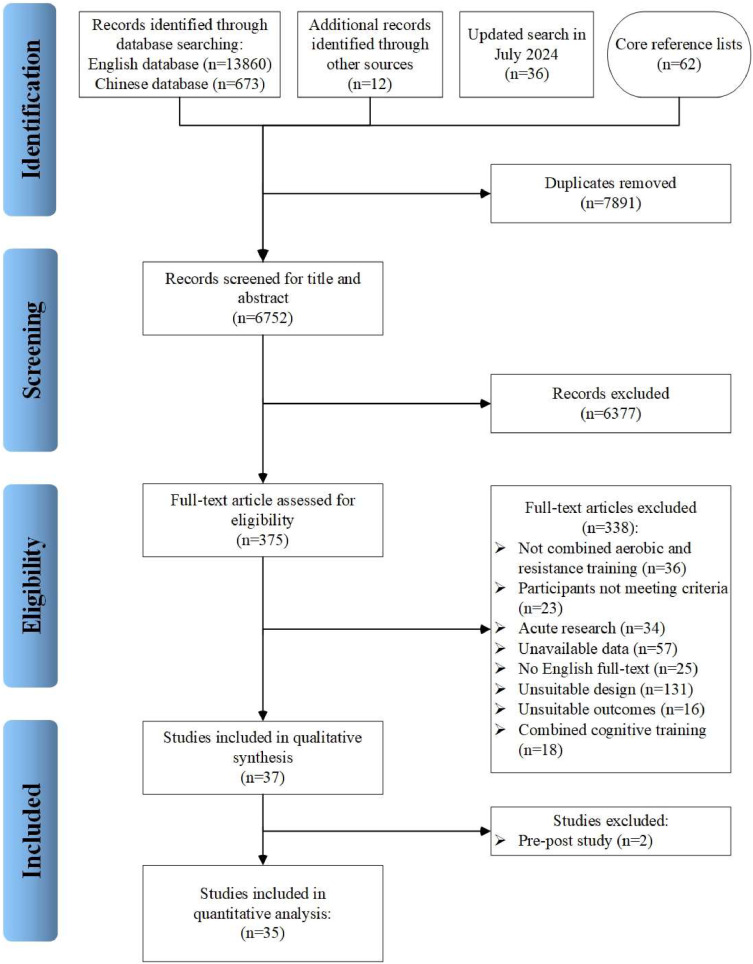
Preferred Reporting Items for Systematic Reviews and Meta-Analysis (PRISMA) flow diagram of each process of the study selection.

The initial search strategy yielded a total of 14,643 records obtained from various electronic databases. After the automatic removal of duplicates and irrelevant records, 375 potential studies remained for further screening. The eligibility was thoroughly assessed to eliminate non-relevant studies, leaving 37 studies that met the inclusion criteria. Two studies (Leach et al., 2016; Marzolini et al., 2013) were conducted by the single-group pre-post study design. Overall, 35 studies (Ansai & Rebelatto, 2015; Arrieta et al., 2020; Barnes et al., 2013; Bell et al., 2019; Bossers et al., 2014; Bossers et al., 2015; Callisaya et al., 2017; Cancela Carral & Ayán Pérez, 2008; Carta et al., 2021; da Silveira Langoni et al., 2019; de Oliveira Silva et al., 2019; de Souto Barreto et al., 2017; Espeland et al., 2017; Fonte et al., 2019; Ghodrati et al., 2023; Henskens et al., 2018; Langlois et al., 2013; Lautenschlager et al., 2008; Lu, 2016; Martínez-Velilla et al., 2021; Munguía-Izquierdo & Legaz-Arrese, 2008; Napoli et al., 2014; Nishiguchi et al., 2015; Nuechterlein et al., 2018; Okumiya et al., 1996; Romberg et al., 2005; Jonatan R Ruiz et al., 2015; Shimada et al., 2018; Sink et al., 2015; Suzuki et al., 2013; Tarazona-Santabalbina et al., 2016; Thaiyanto et al., 2021; Timmons et al., 2018; Vreugdenhil et al., 2012; Williamson et al., 2009) ultimately fulfilled the inclusion criteria. The PRISMA study selection process is illustrated in Fig. 1.

### Characteristics of included studies

The systematic review and meta-analysis included thirty-five RCTs involving 5734 participants aged 22.4 to 92.1 years, published from 1996 to 2022. The sample comprised 2029 males and 3705 females, with a mean age of 72.9 years. The studies were conducted globally, with four (de Oliveira Silva et al., 2019; Ghodrati et al., 2023; Lu, 2016; Thaiyanto et al., 2021) originating from developing countries. Only one study (Lu, 2016) was published in Chinese, whereas the remaining studies were published in English. Participants represented various age groups, including adolescents (Costigan et al., 2016), young adults (Nuechterlein et al., 2023), middle-aged individuals (Ghodrati et al., 2023; Leach et al., 2016; Marzolini et al., 2013; Munguía-Izquierdo & Legaz-Arrese, 2008; Romberg et al., 2005), and the elderly (Ansai & Rebelatto, 2015; Arrieta et al., 2020; Barnes et al., 2013; Bell et al., 2019; Bossers et al., 2014; Bossers et al., 2015; Callisaya et al., 2017; Cancela Carral & Ayán Pérez, 2008; Carta et al., 2021; da Silveira Langoni et al., 2019; de Oliveira Silva et al., 2019; de Souto Barreto et al., 2017; Espeland et al., 2017; Fonte et al., 2019; Henskens et al., 2018; Langlois et al., 2013; Lautenschlager et al., 2008; Lu, 2016; Martínez-Velilla et al., 2021; Napoli et al., 2014; Nishiguchi et al., 2015; Okumiya et al., 1996; Romberg et al., 2005; Jonatan R Ruiz et al., 2015; Shimada et al., 2018; Sink et al., 2015; Suzuki et al., 2013; Tarazona-Santabalbina et al., 2016; Thaiyanto et al., 2021; Timmons et al., 2018; Vreugdenhil et al., 2012; Williamson et al., 2009), with the majority (5382 individuals) classified as older adults. Notably, there is a paucity of research concerning the cognitive health of children and adolescents in the concurrent exercise interventions. Participants were categorised into healthy and clinical populations, with the latter further subdivided into individuals with physiological disorders, mental disorders, cognitive impairments, and age-related cognitive decline.

AET exhibited considerable variability, with regular walking (Arrieta et al., 2020; Bossers et al., 2014; Bossers et al., 2015; Callisaya et al., 2017; da Silveira Langoni et al., 2019; de Oliveira Silva et al., 2019; de Souto Barreto et al., 2017; Espeland et al., 2017; Fonte et al., 2019; Henskens et al., 2018; Lautenschlager et al., 2008; Martínez-Velilla et al., 2021; Marzolini et al., 2013; Nishiguchi et al., 2015; Okumiya et al., 1996; Shimada et al., 2018; Sink et al., 2015; Suzuki et al., 2013; Tarazona-Santabalbina et al., 2016; Vreugdenhil et al., 2012; Williamson et al., 2009) identified as the most common activity, supplemented by running (Costigan et al., 2016; de Oliveira Silva et al., 2019), cycling (Ansai & Rebelatto, 2015; Callisaya et al., 2017; Fonte et al., 2019; Ghodrati et al., 2023; Langlois et al., 2013; Marzolini et al., 2013; Jonatan R Ruiz et al., 2015; Timmons et al., 2018), stepping (Shimada et al., 2018; Tarazona-Santabalbina et al., 2016), swimming, rowing (Callisaya et al., 2017), aerobics (Ghodrati et al., 2023; Lu, 2016; Tarazona-Santabalbina et al., 2016; Thaiyanto et al., 2021), dance-based aerobics (Barnes et al., 2013), and jumping jacks (Costigan et al., 2016). Additionally, SRT primarily utilized four modalities: body-weight (Ansai & Rebelatto, 2015; Bossers et al., 2014; Bossers et al., 2015; Callisaya et al., 2017; Costigan et al., 2016; da Silveira Langoni et al., 2019; Martínez-Velilla et al., 2021; Marzolini et al., 2013; Okumiya et al., 1996; Shimada et al., 2018; Thaiyanto et al., 2021), free weights, weight-lifting machines (Arrieta et al., 2020; Bell et al., 2019; Cancela Carral & Ayán Pérez, 2008; de Oliveira Silva et al., 2019; Fonte et al., 2019; Ghodrati et al., 2023; Henskens et al., 2018; Lu, 2016; Napoli et al., 2014; Jonatan R Ruiz et al., 2015; Timmons et al., 2018), and resistance bands (da Silveira Langoni et al., 2019; Marzolini et al., 2013; Romberg et al., 2005; Jonatan R Ruiz et al., 2015; Tarazona-Santabalbina et al., 2016; Thaiyanto et al., 2021). Four studies (Bell et al., 2019; Costigan et al., 2016; Nuechterlein et al., 2018; Timmons et al., 2018) employed circuit training based on HIIT or MIIT, indicating mixed-order cross-training sessions for AET and SRT. The intensity of AET was adjusted using maximum heart rate (%HRmax), heart rate reserve (%HRR), or maximal oxygen uptake (VO_2_ max), while the intensity of SRT was monitored through the rate of perceived exertion (RPE scales 6–20) (Borg, 1998), percentage of one-repetition maximum (%1RM), or the amount of weight lifted, to assess absolute or relative intensity. Nine studies (Henskens et al., 2018; Lautenschlager et al., 2008; Leach et al., 2016; Lu, 2016; Martínez-Velilla et al., 2021; Nishiguchi et al., 2015; Okumiya et al., 1996; Romberg et al., 2005; Vreugdenhil et al., 2012) did not report load intensity details for both exercise types. These diverse intervention characteristics facilitated a comprehensive analysis of the effects of CT on brain and cognitive health. Exercise frequency ranged from one to eight sessions per week, with session durations spanning 30 to 120 minutes. The majority of interventions lasted less than 16 weeks, with a few extending to 8, 12, or 14 weeks.

In terms of comparators, five studies (Ansai & Rebelatto, 2015; Bossers et al., 2014; Bossers et al., 2015; Costigan et al., 2016; Timmons et al., 2018) compared CT with AET alone (Bossers et al., 2014; Bossers et al., 2015; Costigan et al., 2016; Timmons et al., 2018) or SRT alone (Ansai & Rebelatto, 2015; Costigan et al., 2016; Timmons et al., 2018). Control group included routine activities, no exercise, other exercises, routine care/treatment, social activity, cognitive training, or health education. One study (Nuechterlein et al., 2018) compared CT plus cognitive training to cognitive training alone, while another (Bell et al., 2019) compared CT with nutritional supplementation to CT alone. Furthermore, two studies (Leach et al., 2016; Marzolini et al., 2013) employed a single-group pre-post test design.

The included studies evaluated a range of outcomes related to brain and cognitive health associated with CT. All studies examined the effects of CT on global cognitive function, with seven (Arrieta et al., 2020; Callisaya et al., 2017; Ghodrati et al., 2023; Nishiguchi et al., 2015; Nuechterlein et al., 2018; Jonatan R Ruiz et al., 2015; Suzuki et al., 2013) providing data on structural and biological markers pertinent to brain health. A diverse array of cognitive assessment tools was employed, including the MMSE, 3MSE, MoCA, ACE-R, PASAT, MCCB, SIB-S, and ADAS-Cog. Various tasks were frequently utilized to assess the same cognitive domain within or across studies. A comprehensive description and findings of the study characteristics are presented in Table 2.

**Table 2:** Characteristics of the included studies.

Reference (Country / region)	Year	Population characteristics (age, n, gender, status)	Design	Groups	Intervention		Separate sessions	Frequency and duration	Cognitive task	Key findings
					SRT*	AET*				
Munguía-Izquierdo and Legaz-Arrese (2008) (Spain)	2008	Age: 47.7±8.3 126 F 66.7% FM + 33.3% Healthy	RCT	EG (n=29) : concurrent aerobic and resistance training CG (n=24) : routine activity	Type *: aquatic resistance contraction Intensity: L-M Volume: sets × 1–3 reps × 8–15 Time: 10–20 min	Type: aquatic exercises Intensity ↑: L-V 50–80% HRmax Time: 20–30 min	No	60 min × 3 / wk, 16 wks	PASAT	↑ Cognition MD=6.7 (3.1, 10.3), p=0.004
Bell et al. (2019) (Canada)	2019	Age: 73 ± 6 49 M Healthy	RCT	EG1 (n=18) : concurrent aerobic and resistance exercise + supplement EG2 (n=20) : Concurrent exercise	Type*: weight-lifting machines and free weight Intensity: H 80% 1RM Volume: sets 3 reps 6–8 Intervals 60-s	Type: riding Intensity: H 10 × 60 s HIIT (1: 1) ∼90% HRmax Time: 20-min	Yes	2 SRT + 1 HIIT / wks, 12 wks	MoCA, RAVLT, RT-S, Go-No-Go task	↑ Cognition • ↑MOCA (p = 0.013) • ↑RAVLT (p = 0.047) and↓reaction time (p = 0.002)
Suzuki et al. (2013) (Japan)	2013	Age: 75.3±6.8 49F/51M 50% Healthy + 50% aMCI	RCT	EG (n=50) : concurrent aerobic and resistance exercise CG (n=50) : Health education	Type*: nr Intensity: nr Volume: 20-min	Type: walking Intensity: M ∼60% HRmax Time: 20–30 min	No	90 min × 2 / wk, 6 or 12 months	MMSE, WMS-LM II, DSC, VFT-L+C, SCWT, ADAS-cog, and brain atrophy measurements	↑ Cognition • ↑ MMSE: MD = 0.3 (-0.5, 0.9), p = 0.04 • ↑immediate memory: MD = 2.8 (1.4, 4.2), p < 0.01 • ↑ delayed memory: MD=3.4 (2.0, 4.8), p < 0.01 • ↓brain atrophy: MD = 0.1 (-0.4, 0.7), p < 0.05
Leach et al. (2016)^a^ (Canada)	2016	Age: 50.5±8.7 63 F Breast cancer	Pre-post study	EG (n=63) : BEAUTY program, including aerobic and strength exercise	Type: nr Intensity: nr Volume: nr	Type: nr Intensity: nr Time: nr	No	SRT 1 / wk, AET 2 / wk, 24 wks	FACT-cog	? FACT-cog: MD= 0.9, p > 0.05
Lautenschlager et al. (2008) (Australia)	2008	Age: 68.7±8.6 86F/84M AD	RCT	EG (n=85) : home-based concurrent aerobic and resistance exercise CG (n=85) : routine care	Type: nr Intensity: L Volume: nr	Type: walking Intensity: M Time: nr	nr	50 min × 3 / wk, 18 months	ADAS-Cog, WLDR	↑ Cognition • ↓ADAS-Cog: MD= -0.73 (-1.27, 0.03), p = 0.04
Ansai and Rebelatto (2015) (Brazil)	2014	Age: 82.4±2.4 47F/22M Healthy	RCT	EG1(n=23): aerobic and strength exercise EG2(n=23): resistance training CG (n=23) : routine activity	Type: free weight or body-weight Intensity ↑: V-H RPE 14–17 Volume ↑: reps (up to 15) series (up to 3)	Type *: cycle ergometer Intensity: V 60–85% HRR Time: ∼13-min	No	60 min × 3 / wk, 16 wks	MoCA, CDT, VFT	? MoCA: p > 0.05
Carta et al. (2021) (Italy)	2021	Age: 72.2±4.7 63F/42M Healthy	RCT	EG (n=52) : integrated aerobic and strength exercises CG (n=53) : cultural and educational activities	Type: life movements Intensity: V 60–84% HRR Volume: 45-min	Type: nr Intensity: V 60–84% HRR Time: 45-min	No	60 min × 3 /week, 12 wks	ACE-R	↑ Cognition • ↑ ACE-R: MD = 2.4, p = 0.040 • ↑ Memory: MD = 1.4, p = 0.022 • ↑ visual-spatial skills: MD = 0.6, p = 0.046
Nuechterlein et al. (2018) (USA)	2023	Age: 22.4±3.9 14F/33M Schizophrenia	RCT	EG (n=24) : cognitive training + concurrent circuit training CG (n=23) : cognitive training	Type: MIIT circuit Intensity: nr Volume: 15-min	Type: MIIT circuit Intensity: V 60–80% HRR Time: 30/45 min	No	60 min × 4 /week, 24 wks	MCCB, BDNF, Global Functioning Scale	↑ Cognition • ↑ MCCB: MD = 6.5, p = 0.012 • ↑ work/school functioning: MD = 1.8, p < 0.001 • ? BDNF: MD = 3614, p > 0.05
Nishiguchi et al. (2015) (Japan)	2015	Age: 73.0±4.8 22F/26M Healthy	RCT	EG (n=24) : multimodal exercise CG (n=24) : no exercise	Type*: nr Intensity ↑: nr Volume: nr	Type: walking Intensity: L Time↑: increase daily steps by 15% each month	No	90 min × 1 / wk, 12 wks	MMSE, WMS-LM I/II, TMT, and fMRI	↑ Cognition • ↑ MMSE: MD = 0.8, p = 0.009 • ↑ memory: MD = 4.7 - 5.2, p = 0.008 - 0.009 • ↑ executive function: MD = 13.2, p = 0.018 • ↓ brain activation efficiency: p < 0.001
Cancela Carral and Ayán Pérez (2008) (Spain)	2007	Age: 68.4±3.4 62 F Healthy	RCT	EG (n=27) : water calisthenic exercises combined with strength training CG (n=29) : calisthenic exercises alone	Type: weight-lifting machines Intensity: M 75%1RM Volume: sets × 3 reps × 10	Type: swimming Intensity: H Time: 45-min	No	AET 45 min × 5 + RT × 3 / week, 5 wks	MMSE	↑ Cognition • ↑ MMSE: EG (MD = 2.61, p = 0.03) < CG (MD = 5, p = 0.02)
Bossers et al. (2014) (the Netherlands)	2014	Age: 85.2±6.4 25F/8M Dementia	RCT	EG (n=17) : combined aerobic and strength exercise CG (n=16) : social activity	Type: free weight, body-weight Intensity: M-V RPE 12–15 Weight: body-weight →1.5 kg Volume: sets × 3 reps × 8–30	Type*: outdoor walking Intensity: M-V RPE 12–15 Time: 30-min	Yes	30 min × 5 (2 RT+3 AET) /week, 6 wks	MMSE, FRT, PRT, VMS-F + B, DST-B, VFT, PCT, Stroop task	? Cognition • ? MMSE: ES = 0.13, p = 0.657 • ? FRT: ES = 0.13, p = 0.114 • ↑ VMS-F: ES = 0.68, P = 0.022 • ? VMS-B: ES = 0.03, p = 0.502 • ? DST-B: ES = 0.21, p = 0.576 • ? Stroop task: ES = 0.23, p = 0.721
Bossers et al. (2015) (the Netherlands)	2015	Age: 85.5±5.1 82F/27M Dementia	RCT	EG1 (n= 37) : concurrent aerobic and resistance exercise program EG2 (n= 36) : aerobic exercise alone CG (n=36) : social activity	Type: free weight Intensity ↑: body-weight→1.5kg Volume↑: sets 3 × 2 reps 8→12	Type*: walking Intensity: M-H distances-based Time: 30-min	Yes	30 min × 4 (2 ST+2 AET) / wk, 9 wks	MMSE, Visual memory, Verbal memory	↑Cognition and ↓ cognitive decline EG > CG • ↑ MMSE: ES = 0.48, p < 0.001 • ↑ verbal memory: ES = 0.37, p = 0.032 • ↑ visual memory: ES = 0.46, p = 0.008 • ↑ executive function: ES = 0.37, p < 0.001
Langlois et al. (2013) (Canada)	2012	Age: 72.4±5.7 56F/16M 52.7% Healthy + 47.3% Frail	RCT	EG (n=36) : physical exercise training program CG (n=36) : routine activity	Type: nr. Intensity: M-H Volume: 10 min	Type*: treadmills, recumbent riding, and elliptical Intensity ↑: M→H Time: 10–30 min	No	60 min × 3 /wk, 12 wks	DSC, VFT, VS, RAVLT	↑ Cognition • ↑ executive functions (p = 0.039) • ↑ processing speed (p = 0.014) ↑ working memory: (p = 0.035)
Williamson et al. (2009) (USA)	2009	Age: 77.4±4.3 72F/30M Healthy	RCT	EG(n=50): physical activity, including aerobic, strength exercises CG(n=52): health education	Type: nr. Intensity: M Volume: nr.	Type: walking Intensity: M 60%-70% HRmax Time: 40–60 min	nr	≥40–60 min × 3/2+3 /wk, 48 wks	3MSE, DSST, RAVLT, and modified Stroop test	? No improvement • ↑ DSST: MD = 0.56, p < 0.05 • ? RAVLT (p > 0.05) • ? 3MSE (p > 0.05) ? modified Stroop Test (p > 0.05)
Sink et al. (2015) (USA)	2015	Age: 78.9±5.2 999F/477M 189 MCI or dementia	RCT	EG (n=735) : physical activity intervention CG (n=741) : successful aging health education	Type: free weight Intensity: M Volume: 10-min	Type*: walking Intensity: M Time: 30-min	No	∼60 min × 2+3–4 /wk, 96 wks	DSC, HVLT-R, 3MSE	? No improvement • ? DSC (p = 0.97) ? HVLT-R (p = 0.84)
Thaiyanto et al. (2021) (Thailand)	2020	Age: 67.8±2.5 40 F MCI + AD	RCT	EG (n=20) : multicomponent exercise including aerobic and resistance exercises CG (n=20) : health education	Type*: resistance band or body-weight Intensity ↑: M increase by repetitions or load RPE ≤13–14 Volume↑: 15-min	Type: aerobics Intensity ↑: M-V RPE 13–14 Time: 15-min	No	60 min × 3 /wk, 12 wks	ADAS-Cog, TMT, Stroop test	↑ Attention and executive function TMT-A (p < 0.05)
de Oliveira Silva et al. (2019) (Brazil)	2019	Age: 77.2±7.0 27F/19M MCI + AD	RCT	EG (n=24) : multimodal exercise program (including aerobic and resistance) CG (n=14) : no exercise	Type: weight-lifting machines Intensity: M Volume: sets 3 reps 8–12	Type*: treadmill running or walking Intensity: V 70% VO_2_max / 80% HRmax Time: 30-min	No	60 min × 2 /wk, 12 wks	MMSE, CDT, VFT, ST3	↑ verbal fluency ↑ VFT (p = 0.05)
Napoli et al. (2014) (USA)	2014	Age: 69 ± 4 72F/35M Healthy	RCT	EG (n=54) : exercise (+ diet) program CG (n=53) : health education (+diet)	Type: weight-lifting machines Intensity ↑: M→H ∼65 → ∼80% 1RM Volume: 30 min sets 1–3 reps 6–12	Type*: nr Intensity ↑: V-H ∼65% → 70–85% HRpeak Time: 30-min	No	90 min × 3 /wk, 52 wks	3MSE, WLF, TMT A+B	↑ Cognition • ↑ 3MSE: MD = 1.7, p = 0.0001–0.04 • ↑ WLF: MD = 4.1, p = 0.001 • ↑ TMT-A: MD = 211.8, p = 0.001 ↑ TMT-B: MD = 221.8, p = 0.001
Shimada et al. (2018) (Japan)	2018	Age: 71.6±5.0 154F/154M MCI	RCT	EG (n=154) : combined activity program, including aerobic exercise, muscle strength training CG (n=154) : health education	Type*: free weight or body-weight Intensity: nr Volume: 20-min	Type: stepping and walking Intensity: M-V 60–80% HRmax Time: 25-min	No	90 min × 1 /wk, 40 wks	MMSE, WMS-LM II, RAVLT, VFT-letter, VFT-category, TMT	↑ Cognition • ↑ MMSE (p = 0.012) • ↑ WMS-LM II (p = 0.004) • ? RAVLT (p = 0.352) • ↑ VFT-letter (p < 0.001) ↑ VFT-category (p = 0.002)
Timmons et al. (2018) (Ireland)	2018	Age: 69.3±3.5 39F/45M Healthy	RCT	EG1 (n=21) : aerobic exercise alone EG2 (n=21) : resistance exercise alone EG3 (n=21) : concurrent exercise CG (n=21) : no exercise	Type*: Circuit-based weight-lifting machines and free weight Intensity: M ∼60% 1RM Volume: reps for 1 min rounds × 2 exercise × 6 recovery × 30s	Type: MIIT-based cycle ergometer Intensity: V ∼80% HRmax Time: 12-min sets × 1.5 recovery for 1-min	No	40 min × 3 / wk, 12 wks	MoCA	↑ Cognition ↑ MoCA (p < 0.001)
Henskens et al. (2018) (the Netherlands)	2018	Age: 85.7±5.6 67F/20M Dementia	RCT	EG (n=22) : concurrent exercise training CG (n=22) : social activity	Type: weight-lifting machines or free weight Intensity ↑: nr weight ↑ Volume: sets × 3 reps 8→15	Type: outdoor walking Intensity ↑: nr optimal walking speed Time: 30–45 min	Yes	30–45 min × 3 /wk, 6 months	MMSE, SIB-S, executive function: DST-B, CFT, Go-No-Go test,	• ? MMSE (p = 0.83) • ? SIB-S (p = 0.85) • ? DST-F (p = 0.13) • DST-B (p = 0.88) • ↑ EF (p = 0.007) • ↑ CFT (p = 0.004) ↑ Go-No-Go test (p = 0.04)
Espeland et al. (2017) (USA)	2016	Age: 70–89 899F/477M Diabetes	RCT	EG (n=735) : aerobic, resistance training, and flexibility exercises CG (n=741) : health education	Type: free weight Intensity: V RPE 15–16 Volume: 10-min	Type*: walking Intensity: M RPE 13 Time: 30-min	No	∼60 min × 2 CB + 3–4 HB /wk, 26 wks	3MSE, DSC, HVLT-R	↑ Global cognition • ↑ 3MSE (p = 0.02) • ? DSC (p = 0.87) • ↑ HVLT-R (p = 0.005) ? EF (p = 0.70)
Costigan et al. (2016) (Australia)	2016	Age: 15.8 ± 0.6 20F/45M Healthy	RCT	EG1 (n=21) : aerobic exercise program EG2 (n=22) : concurrent resistance and aerobic program CG (n=22) : physical education and usual lunchtime activities	Type: HIIT-based body-weight Intensity: V ≥85% HRmax Volume: reps for 30-s, work-to-rest ratio: 30:30	Type: HIIT shuttle runs, jumping jacks, and skipping Intensity: V ≥85%HRmax Time: 8–10 min, work-to-rest ratio: 30:30	No	8–10 min × 3/5 (3–4 ST+1 AET) / wk, 8 wks	TMT	↑ Executive function (p < 0.05)
Romberg et al. (2005) (Finland)	2005	Age: 43.9±6.7 61F/34M MS	RCT	EG (n=47) : concurrent resistance and aerobic training CG (n=48) : no intervention	Type: resistance band Intensity: nr Volume: nr	Type: nr. Intensity: nr Time: nr.	Yes	4–6 (3–5 ST+1AET) /wk, 26 wks	PASAT	༟No improvement (p = 0.379)
Vreugdenhil et al. (2012) (Australia)	2012	Age: 74.1(51–89) 24F/16M AD	RCT	EG (n=20) : home-based concurrent exercise CG (n=20) : routine treatment	Type: nr Intensity: nr Volume: nr	Type: walking Intensity: nr. Time: ≥30 min	No	30 min × daily, 4 months	ADAS-Cog, MMSE	↑ Cognition • ↑ MMSE: MD = 2.6, p < 0.001 ↓ADAS-Cog: MD = -7.1, p <0.001
Arrieta et al. (2020) (Spain)	2020	Age: 84.9±6.8 79F/33M Healthy	RCT	EG (n=43) : multicomponent exercise program, including strength and walking CG (n=45) : routine activity	Type*: free weight or weight-lifting machines Intensity ↑: L-M 40–70%1RM →65–70%1RM Volume: sets 1–2 reps 7–12 exercise × 3–5	Type: walking Intensity ↑: M CR10 scale 5–6 Time ↑: 5→22 min	No	45 min × 2 / wk, 6 months	MoCA, RAVLT, TMT-A, WAIS-IV, VFT, SFT	↑ Cognition • ↑ MoCA (p = 0.003) • ↑ Symbol search (p = 0.049) • ? TMT-A (p = 0.836) • ↑ WAIS-IV (p < 0.05) • ↑ VFT (p < 0.05) • ↑ SFT (p < 0.05) • ? serum BDNF levels (p > 0.05)
de Souto Barreto et al. (2017) (France)	2017	Age: 87.6±5.5 77F/14M Dementia	C-RCT	EG (n=47) : group-based exercise CG (n=50) : social activity	Type*: free weight Intensity ↑: M Volume↑: 10–15 min	Type: walking Intensity ↑: M Time: 20-min	No	60 min × 2 / wk, 24 wks	MMSE	? No improvement MMSE (p = 0.43)
Jonatan R Ruiz et al. (2015) (Spain)	2015	Age: 92.1±2.1 32F/8M	RCT	EG (n=20) : aerobic and resistance exercises CG (n=20) : routine care	Type: resistance band or weight-lifting machines Intensity: L→M↑ Volume: sets 2–3 reps 8–10 interval 1–2 min	Type*: cycle-ergometer Intensity: L-M RPE 10–12 Time: 10–15 min	No	40–45 min × 3 / wk, 8 wks	MMSE, ACE, BDNF, EGF, TNF-α	? No improvement • MMSE (p = 0.97)
Ghodrati et al. (2023) (Iran)	2022	Age: 57.8±1.5 21 F Diabetes	RCT	EG (n=12) : concurrent training CG (n=9) : no exercise	Type: weight-lifting machines Intensity ↑: M→H 65%→85% 1RM Volume↑: sets 3 reps 6–12 intervals for 1-min Time: 30-min	Type*: aerobics, elliptical trainer and stationary bicycle Intensity ↑: M-V 55%→75%HRR Sets 4 Time: 20-min	No	∼65 min × 3 / wk, 12 wks	MoCA, DSST, DST-F	? Cognition • MoCA (p = 0.05) • DSST (p = 0.33) • DST-F (p = 0.07) • ༟BDNF or irisin levels (p > 0.05)
Barnes et al. (2013) (USA)	2013	Age: 73.4±5.9 79F/47M Healthy	RCT	EG (n=31) : concurrent aerobic and strength exercise CG (n=32) : other exercises	Type: nr Intensity: nr Volume: 10-min	Type*: aerobic dance Intensity: V 60–75% HRpeak Time: 30-min	No	60 min × 3 / wk, 12 wks	3MSE, RAVLT, DSST, TMT-A+B, EFT, UFOV,	? Cognition • ↑3MSE: MD = 0.16 (0.05, 0.26), p < 0.001 • RAVLT: p= 0.38, p = 0.93 • DSST: p = 0.71 • TMT-A: p = 0.24 • TMT-B: p = 0.31 • EFT: p = 0.51, p = 0.60 • UFOV processing speed (p = 0 0.65) divided attention (p = 0.05) ↑selective attention (p = 0.02)
Martínez-Velilla et al. (2021) (Spain)	2021	Age: 86.5±5 53F/50M Diabetes	RCT	EG (n=54) : multicomponent exercise CG (n=49) : usual care	Type: body-weight or free weight Intensity ↑: nr Volume: sets 3 reps 12	Type: walking Intensity: L Time: 20-min	No	40 min × 5–7 / wk, 12 wks	MMSE	↑ Cognition MMSE: MD = 1.7, p <0.001
Callisaya et al. (2017) (Australia)	2017	Age: 66.2±4.9 24F/26M Diabetes	RCT	EG (n=26) : concurrent aerobic and resistance training CG (n=24) : other exercises	Type*: body-weight, weight-lifting machine or free weights Intensity ↑: V-H RPE 14–17 Volume: 30-min sets 3 reps 8–12	Type: stationary cycling, cross trainer, rower or treadmill walking Intensity ↑: M→V→H RPE 12→16 Time: 30-min	No	60 min × 3 / wk, 6 months	TMT, DSC, WMS-LM Ⅲ, COWAT, HVLT-R, RCF, DST-F+B, and Stroop test	? Cognition • MMSE: MD = 0.14 • EF: MD = -3.8 (-7.1, -0.5) • processing speed: MD = -8.7 (-17.9, 0.5) • DSC: MD = +2.2 (0.2, 4.2) • HVLT-R: MD_1_ = 0.7 (-0.9, 2.2), MD_2_=0.1 (-0.7, 0.9) • COWAT: MD_1_ = 3.1 (0.4, 5.7), MD_2_ = 0.9 (-2.6, 0.8) • ↑ white matter integrity, hippocampal and total brain volumes
da Silveira Langoni et al. (2019) (Brazil)	2019	Age: 72.3±7.9 40F/12M MCI	RCT	EG (n=26) : group-based aerobic and strength training program CG (n=26) : no exercise	Type: free weight, resistance band, or body-weight Intensity ↑: nr Volume ↑: 30-min sets 2 reps 10–15	Type: walking Intensity: 60–75% HRmax Time ↑: 20-min → 25-min → 30-min	No	∼60 min × 4 / wk, 6 months	MMSE	↑ Global cognition ↑ MMSE: MD = 3.1 ± 2.4, p < 0.001
Marzolini et al. (2013) (Canada)	2013	Age: 63.6±13.5 11F/30M Post-stroke	Pre-post study	EG (n=41) : concurrent aerobic and resistance exercise	Type: free weight, resistance band, or body-weight Intensity: M 50–60% 1RM RPE 13–14 Volume: Sets based on RPE reps 10–15	Type *: walking, cycling Intensity: L-V 40–70% VO_2_peak RPE 11–16 Time: 20–60 min	Unclear	90 min × 1+5 (home-based:4 AET+1–2 ST) / wk, 6 months	MoCA	↑ Cognition • ↑ MoCA: MD = 1.5, p < 0.001 • ↑ attention: MD = 0.5, p =0.03 ↑ executive function: MD = 0.5, p = 0.002
Tarazona-Santabalbina et al. (2016) (Spain)	2016	Age: 80.0±3.7 54F/46M Healthy	RCT	EG (n=51) : multicomponent exercise program CG (n=49) : routine care	Type: resistance band Intensity ↑: L→M 25→75%1RM Volume: sets 1–3 reps 8–30	Type*: walking, aerobics, step Intensity ↑: L-M 40–65% HRmax Time: 40-min	Yes	40 min × 5 (2ST + 3AET) / wk, 14 wks	MMSE	↑ Global cognition MMSE: MD = 2.4, p = 0.025
Fonte et al. (2019) (Italy)	2019	Age: 78±6 55 F/32M 31% MCI + 69% AD	RCT	EG (n=27) : endurance and resistance training CG (n=30) : no intervention	Type: isotonic ergometers weight-lifting machines Intensity ↑: H 85%1RM Volume ↑: sets 3 reps 12	Type*: cycling, treadmill walking, arm cranking Intensity ↑: M-V 70% HRmax Time: 45-min	No	90 min × 3 / wk, 24 wks	MMSE, FAB, TMT-A+B, RBMT, DCT , ADAS-Cog	↑ Global cognition and ↓cognitive decline • MMSE: MD = 0% • TMT-A: MD = -12.4% • TMT-B: MD = -7.8% • RBMT: MD = +6.9%
Okumiya et al. (1996) (Japan)	1996	Age: 78.8±4.6 24F/18M Healthy	RCT	EG (n=21) : multicomponent exercise CG (n=21): no exercise	Type: body-weight Intensity: L Volume: nr	Type*: walking Intensity: L Time: 50-min	No	60 min × 2 / wk, 24 wks	MMSE, HDS-R, VCP-test	? No improvement • MMSE (p > 0.05) • HDS-R (p > 0.05) VCP-test (p > 0.05)
Lu (2016) (China)	2016	Age: 74.5±4.6 70F/78M MCI	RCT	EG (n=74) : concurrent resistance and aerobic training CG (n=74): rehabilitation training and routine health education	Type: free weight or weight-lifting machines Intensity: M-H Volume: nr	Type: aerobics Intensity: M-H Time: 40-min	No	60 min × 5 / wk, 12 months	MoCA	? No improvement • MoCA (p > 0.05)

RAVLT: the Rey Auditory Verbal Learning Test, PASAT: Paced Auditory Serial Addition Test, MMSE: Mini-mental state examination, 3MSE: modified Mini-mental state exam, MoCA: Montréal Cognitive Assessment, SCWT: Stroop color word test, WLDR: word list delayed recall, CDT: clock drawing test, VFT: verbal fluency test, VFT-L+C: letter and categorical verbal fluency test, CFT: category fluency test, SFT: semantic fluency tests, WLF: word list fluency test, RT-S: simple reaction time task, RT-C: choice reaction time task, MCCB: MATRICS consensus cognitive battery, fMRI: functional magnetic resonance imaging, FRT: faces recognition test, PRT: pictures recognition test, DSC: digit symbol coding test, DCT: digit cancellation test, DSST: digit symbol substitution test, DST-F+B: digit span test forward and backward, ADAS-Cog: Alzheimer Disease Assessment Scale-Cognitive Subscale, FACT-Cog: Functional Assessment of Cancer Therapy - Cognitive Function; ACE-R: Addenbrooke's Cognitive Examination Revised, TMT-A+B: Trail making part A+B, RNG: random number generation task, VMS-F+B: visual memory span forward and backward, PCT: picture completion test, VS: visual-spatial abilities, EFT: Eriksen Flanker test, UFOV: Useful field of view, RCF: Rey complex figure, COWAT: controlled oral word association test, FAB: frontal assessment battery, VCP-test: Visuo-spatial cognitive performance test, HDS-R: Hasegawa Dementia Scale-Revised, RBMT: Rivermead behavioral memory test, HVLT-R: Hopkins Verbal Learning Test-Revised, SIB-S: severe impairment battery – short form, WMS-LM II: logical memory subtest of the Wechsler memory scale-revised, WAIS-III: Wechsler Adult Intelligence Scale – 3rd Edition, BDNF: brain - derived neural factor, ACE: angiotensin – converting enzyme, EGF: epidermal growth factor, TNF-α: tumor necrosis factor-α;

AET: aerobic / endurance / cardiovascular / cardiorespiratory exercise training, SRT: strength / resistance exercise training, EG: experimental group, CG: control group, wk: week, HIIT: high-intensity interval training, MIIT: moderate-intensity interval training, BEAUTY program: Breast cancer patients Engaging in Activity while Undergoing Treatment, HRmax / HRpeak: maximum heart rate, %HRR: heart rate reserve =HRmax −resting HR, %VO_2_max: maximal oxygen uptake, MAP: maximal aerobic power, 1RM: one-repetition maximum, Reps: number of repetitions, RPE: Rate of Perceived Exertion, based on Borg RPE scale, including category scale (6–20) and category-ratio scale (0–10); METs: Metabolic Equivalent, 1METs=3.5ml O_2_/kg/min, S: sedentary, L: light, M: moderate, V: vigorous, H: high, MD: mean difference, ES: effect size, Cohen's d;

FM: Fibromyalgia syndrome, (a)MCI: (amnestic) mild cognitive impairment, AD: Alzheimer's disease, MS: multiple sclerosis;

* indicating that this exercise was performed before the other exercise

↑ indicating a progressive load from baseline to post-intervention

a indicating study was conducted with pre-post design

### Risk of bias

**Fig. 2:**
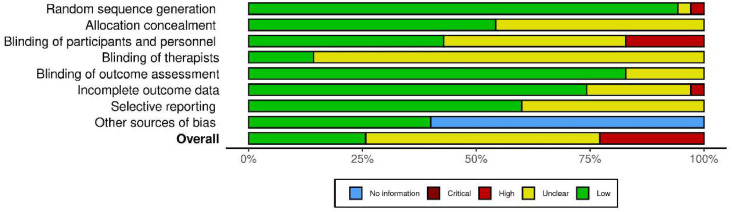
Analysis of the risk of bias indicated by percentages of assessed biases in included studies.

The risk of bias assessment is summarised in **Supplementary file S3** and Fig. 2. Most studies exhibited a low risk for random sequence generation and outcome assessment blinding. However, allocation concealment and participant blinding were frequently unclear or high-risk. Incomplete outcome data and selective reporting presented moderate risks, while other sources of bias were minimal.

### Overall analyses and quality of evidence

**Fig. 3:**
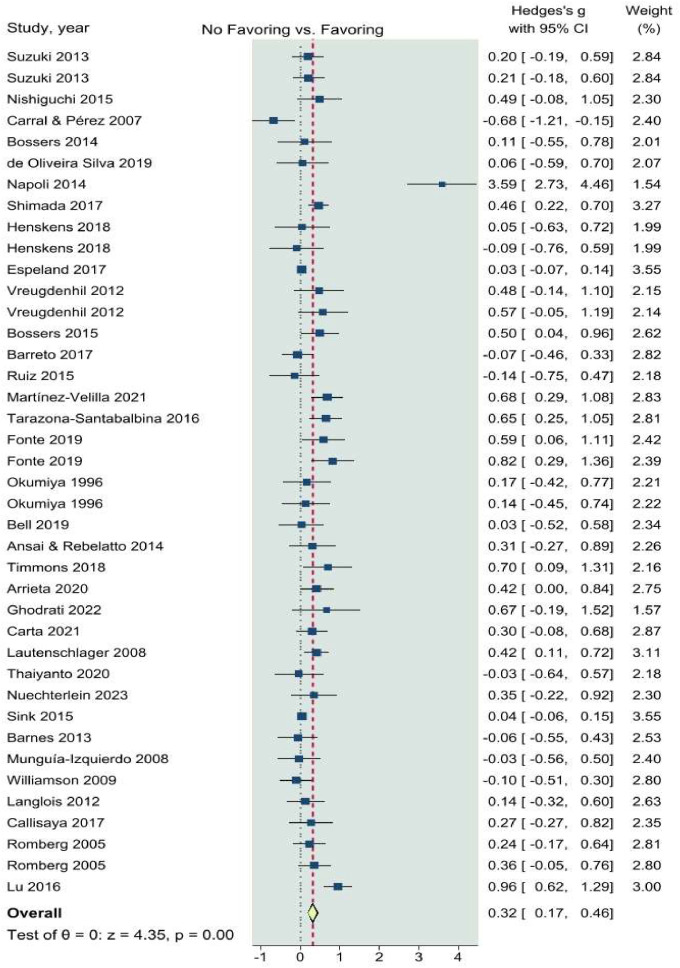
Forest plot of the overall effects of concurrent training on global cognition. CI, confidence interval.

The forest plot of the effects of CT interventions on global cognition is presented in Fig. 3. A total of k=41 dependent ES were included in the overall analysis. The overall ES revealed by the meta-analysis was significant and positive (SMD = 0.32; 95% CI: 0.17–0.46, p < 0.001). Twelve statistically significant results (p < 0.05) are shown in **Supplementary file S4**, with nine having p-values below 0.025, all included in the p-curve. The p-curve analysis revealed significant effects with a power estimate of 91%, supporting the research hypothesis. However, substantial heterogeneity was noted (Q_40_ = 178.6, p < 0.0001, tau² = 0.21, I² = 86.0%) (Higgins et al., 2003).

**Fig. 4:**
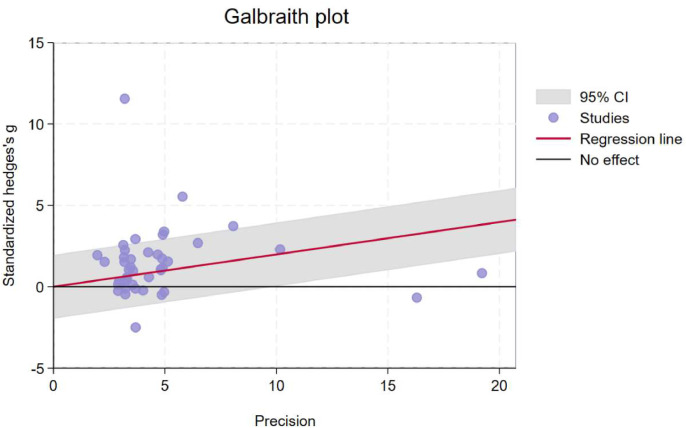
Galbraith plot for assessing the heterogeneity in meta-analysis.

The GRADE analysis indicated that the overall quality of evidence was moderate. Key limitations were identified, including variability across studies (inconsistency) and the potential for publication bias. The funnel plot of included studies is displayed in **Supplementary file S5**. An examination of the studentized residuals revealed that one study (Napoli et al., 2014) had a value larger than ± 3.23 and may be a potential outlier in the context of this model. According to the Cook's distances, two studies (da Silveira Langoni et al., 2019; Napoli et al., 2014) could be considered to be overly influential. Neither the Begg rank correlation nor the Egger's regression test indicated any funnel plot asymmetry (p = 0.76 and p = 0.08, respectively) and potential publication bias. Fig. 4 showed that Galbraith plot uncovered significant heterogeneity in the study results; therefore, moderator analyses, subgroup analysis and meta regression were conducted.

### Moderator analyses

To investigate potential sources of variance in the effects of CT on global cognitive function, moderator analyses were conducted using distinct models. The findings from this analysis are summarised in Table 3. For each moderator analysis, the omnibus test yielded significant results, and the 95% CI for all factors overlapped.

**Table 3:** Moderator analysis of effects of concurrent training on cognitive function outcomes.

Moderator	k	Hedges' g (95%CI)	Q statistic	P value
**Participants moderators**				
Age, year				
18–65	4	0.24 (0.00 to 0.48)	Q = 145.76; I^2^=83.38; p<0.01	0.099
>65	31	0.33 (0.14 to 0.51)		0.004
Health status				
Healthy population	12	0.59 (-0.03 to 1.20)	Q = 151.13; I^2^=84.57; p<0.01	0.063
Clinical population	20	0.28 (0.11 to 0.46)		<0.001
Both	4	0.05 (-0.05 to 0.15)		0.33
**Exercise moderators**				
Same session				
Yes	30	0.38 (0.10 to 0.66)	Q = 158.82; I^2^=82.23 p<0.01	0.007
No	5	0.39 (0.09 to 0.68)		0.011
Additional exercises				
Yes	18	0.26 (0.10 to 0.42)	Q = 182.96; I^2^=82.54; p<0.01	0.001
No	17	0.51 (-0.05 to 1.07)		0.066
Configuration				
AET first	13	0.45 (-0.05 to 0.96)	Q = 147.56; I^2^=80.45; p<0.01	0.08
SRT first	10	0.33 (0.13 to 0.48)		<0.001
Circle	3	0.54 (0.17 to 0.91)		0.005
Separation	4	0.23 (-0.06 to 0.51		0.119
Unclear	8	0.25 (-0.05 to 0.55)		0.106
Duration, min				
Short (30–45)	12	0.28 (0.04 to 0.53)	Q = 145.63; I^2^=82.75; p<0.01	0.024
Medium (60)	17	0.21 (0.06 to 0.35)		0.006
Long (90)	5	1.03 (-0.16 to 2.22)		0.089
Frequency, day/week				
Low (1–2)	8	0.29 (0.13 to 0.48)	Q = 146.40; I^2^=81.71; p<0.01	0.002
Medium (3–4)	20	0.32 (0.02 to 0.63)		0.036
High (≥5)	8	0.42 (0.14 to 0.70)		0.004
Length, week				
Short (4–12)	13	0.28 (0.11 to 0.44)	Q =182.96; I^2^=82.54 p<0.01	0.001
Medium (13–26)	16	0.21 (0.05 to 0.37)		0.011
Long (>26)	7	0.75 (-0.13 to 1.63)		0.093
**Cognitive moderators**				
Cognitive tasks				
MMSE	15	0.33 (0.19 to 0.47)	Q =182.96; I^2^=82.54 p<0.01	<0.001
3MSE	5	0.53 (-0.93 to 1.99)		0.479
ADAS-Cog	3	0.37 (0.12 to 0.61)		0.004
GCS	2	0.05 (-0.05 to 0.15)		0.316
MoCA	6	0.53 (0.23 to 0.84)		0.001
Other cognitive tests	5	0.22 (0.01 to 0.43)		0.043
**Comparison moderators**				
Comparison type				
Health education	9	0.68 (-0.39 to 1.75)	Q = 143.08; I^2^=85.10 p<0.01	0.215
No exercise	7	0.44 (0.19 to 0.69)		0.001
Other exercises	4	0.07 (-0.23 to 0.38)		0.643
Routine activity	4	0.30 (0.02 to 0.57)		0.033
Routine treatment / care	5	0.45 (0.05 to 0.85)		0.027
Social activity	4	0.18 (-0.20 to 0.55)		0.358

k: number of effect sizes, CI: confidence intervals, min: minutes, AET: aerobic / endurance / cardiovascular / cardiorespiratory exercise training, SRT: strength / resistance exercise training.

***p < 0.001, **p < 0.01, *p < 0.05

#### Participants moderators

Participants aged over 65 exhibited significant effects (g = 0.33, 95% CI: 0.14 to 0.51; p < 0.05), whereas those aged 18 to 65 did not (p = 0.099), indicating greater cognitive benefits from exercise in older adults. Clinical populations demonstrated significant effects (g = 0.28, 95% CI: 0.11 to 0.46; p < 0.001), while healthy populations exhibited a non-significant trend (p = 0.063), and mixed-status populations did not show significant effects (p = 0.33).

#### Exercise moderators

Both exercises performed within the same session (g = 0.34, 95% CI: 0.10 to 0.66; p = 0.007) and CT supplemented with additional interventions (g = 0.26, 95% CI: 0.10 to 0.42; p = 0.001) exhibited significant effects. SRT performed first (g = 0.33, 95% CI: 0.13 to 0.48; p < 0.001) and circuit training (g = 0.54, 95% CI: 0.17 to 0.91; p = 0.005) exhibited significant effects, whereas AET performed first (p = 0.08) and training separation (p = 0.119) were non-significant. Both short-duration (g = 0.28, 95% CI: 0.04 to 0.53; p = 0.024) and medium-duration (g = 0.21, 95% CI: 0.06 to 0.35; p = 0.006) interventions demonstrated significant improvements. Exercise frequency yielded significant estimates across all three categories: low (g = 0.29, 95% CI: 0.13 to 0.48; p = 0.002), medium (g = 0.32, 95% CI: 0.02 to 0.63; p = 0.036), and high (g = 0.42, 95% CI: 0.14 to 0.70; p = 0.004). Medium-length interventions (13–26 weeks) demonstrated a significant ES (g = 0.21, 95% CI: 0.05 to 0.37; p = 0.011). Short interventions (4–12 weeks) exhibited a highly significant effect (g = 0.28, 95% CI: 0.11 to 0.44; p = 0.001). In contrast, long interventions (>26 weeks) exhibited a large effect size (g = 0.75, 95% CI: -0.13 to 1.63) but did not achieve statistical significance (p = 0.093), suggesting variability in their efficacy or the necessity for larger sample sizes to attain significance.

#### Cognitive moderators

Cognitive tasks varied in effectiveness, with the MMSE, ADAS-Cog, and MoCA demonstrating significant effects (p < 0.001, p = 0.004, and p = 0.001, respectively), whereas the 3MSE and GCS did not achieve significance (p = 0.479 and p = 0.316, respectively). These findings suggest that certain assessments are more sensitive to exercise-related cognitive changes.

#### Comparison moderators

Comparison types significantly influenced intervention outcomes: Health education control exhibited a large ES (g = 0.68), although it was non-significant (95% CI: -0.39 to 1.75; p = 0.215); no exercise control demonstrated a significant moderate ES (g = 0.44, 95% CI: 0.19 to 0.69; p = 0.001); other exercise controls yielded a small and non-significant ES (g = 0.07, 95% CI: -0.23 to 0.38; p = 0.643); routine activity control indicated a significant ES (g = 0.30, 95% CI: 0.02 to 0.57; p = 0.033); routine treatment/care control exhibited a significant moderate ES (g = 0.45, 95% CI: 0.05 to 0.85; p = 0.027); social activity control was non-significant (g = 0.18, 95% CI: -0.20 to 0.55; p = 0.358). These results underscore the significance of designing appropriate comparison conditions in intervention studies.

### Meta‑regression analysis and subgroup analyses

**Fig. 5:**
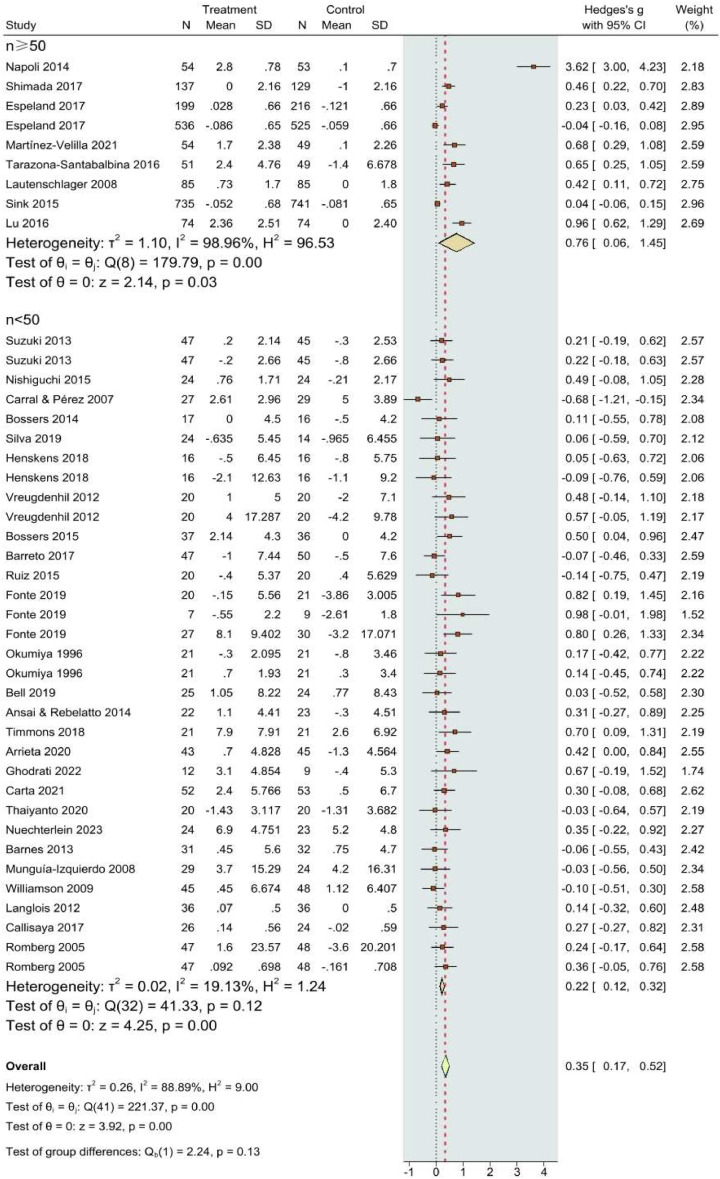
Effects of concurrent training on cognitive outcomes: subgroup analysis by single-group sample sizes. CI, confidence interval; IV, inverse variance; SD, standard deviation.

**Fig. 6:**
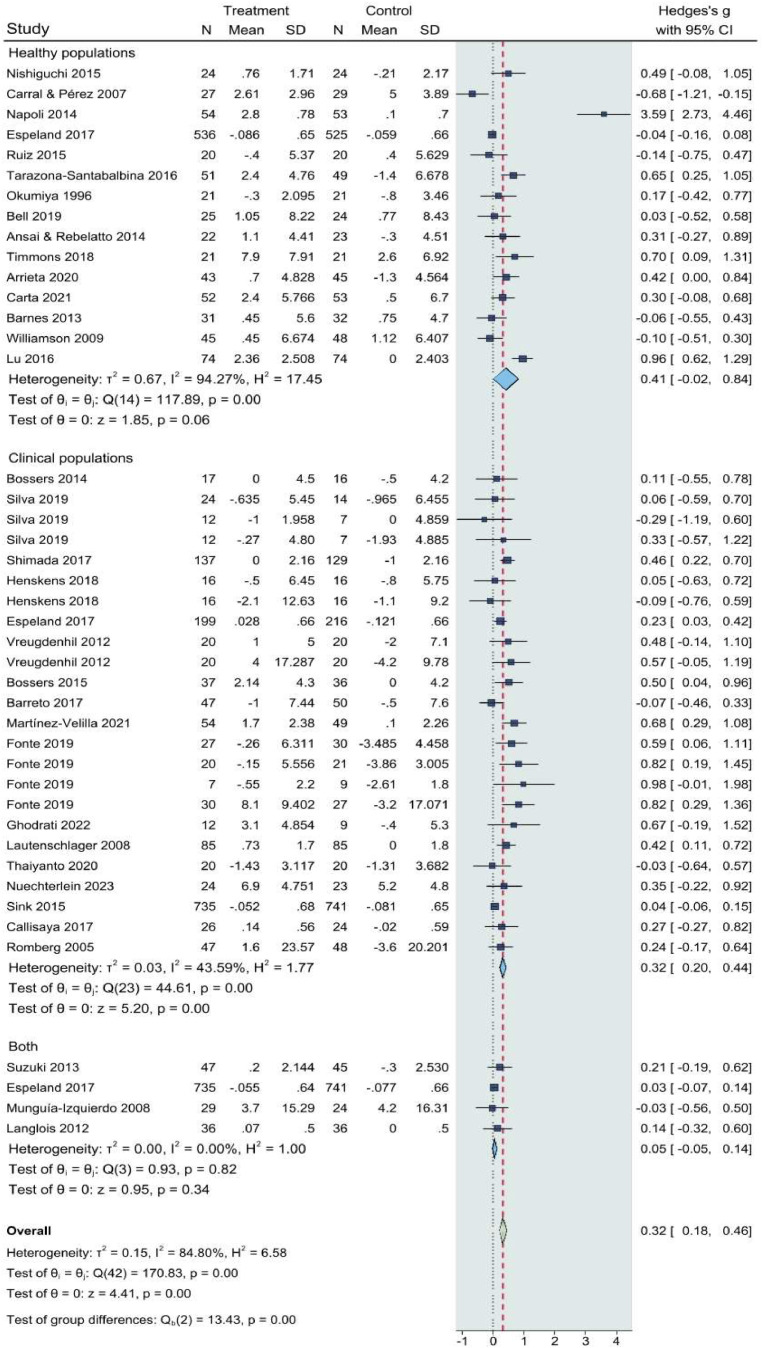
Effects of concurrent training on cognitive outcomes: subgroup analysis by participants’ health status. CI, confidence interval; IV, inverse variance; SD, standard deviation.

The results of the univariate moderator analyses are presented in Table 4. The meta-regression analysis indicated that single-group sample size (R^2^ = 0.51, p = 0.031) and health status (R^2^ = 0.495, p = 0.045) significantly contributed to the heterogeneity of intervention effects. Consequently, subgroup analyses were conducted on these variables. The results of the predefined subgroup analyses are illustrated in Fig. 5, Fig. 6. The subgroup analyses indicated that a sample size of n ≥ 50 and healthy populations were potential sources of heterogeneity (Q = 117.89 - 179.79, p < 0.01; I² = 94.27 - 98%), suggesting that the magnitude of treatment effects or overall health of participants may be influenced by the size of the study population.

**Table 4:** Meta-regression analysis for potential heterogeneity.

Covariates	k	SE (95% CI)	R^2^	p value
Age (18–65 years, >65 years)	36	0.25 (-0.47 to 0.54)	0.03	0.69
Gender (Female>50%)	33	0.24 (-0.34 to 0.63)	0.144	0.550
Health status (Healthy vs. Clinical)	42	0.24 (0.01 to 0.98)	0.495*	0.045
Single-group sample size (n>50 or n≤50)	39	0.20 (0.05 to 0.86)	0.51*	0.031
Comparison type	41	0.08 (-0.15 to 0.19)	-0.017	0.864
Additional exercise (Yes or No)	47	0.38 (-0.09 to 1.55)	0.961	0.079
Length (<16 wks, 16–26 wks, ≥26 wks)	47	0.25 (-0.35 to 0.74)	0.637	0.16
Frequency (1–2, 3–4, ≥5)	37	0.36 (-1.14 to 0.44)	-0.045	0.893
Same session (Yes or No)	36	0.46 (-0.89 to 1.10)	0.076	0.93
Configuration (SRT first or AET first)	27	0.24 (-0.44 to 0.61)	-0.149	0.623
Cognitive tasks	48	0.11 (-0.21 to 0.26)	-0.161	0.292

k: number of effect sizes, SE: standard error; CI: confidence intervals; R^2^: regression coefficient; wks: weeks; AET: aerobic / endurance / cardiovascular / cardiorespiratory exercise training, SRT: strength / resistance exercise training.

***p < 0.001, **p < 0.01,

⁎ p < 0.05

## Discussion

This meta-analysis represents the first comprehensive evaluation of the effects of CT on cognitive health, incorporating data from 35 RCTs involving 5734 participants. The main findings indicate that CT effectively enhances overall cognitive function, though its effects are not superior to those of single-modality training. Specifically, older adults (>65 years) and clinical populations demonstrated the greatest cognitive benefits from CT interventions compared to younger adults (18–65 years) and healthy populations. In terms of exercise configurations, the most pronounced cognitive effects were observed with supplemental interventions, sessions lasting 30 to 60 minutes, intervention durations of 4 to 26 weeks, and the prioritization of SRT over AET (intra-session SRT prior to AET) within a single session. Furthermore, meta-regression analysis suggested that heterogeneity could stem from sample size and participants’ health status, with healthy populations and sample sizes of ≥50 partially accounting for the observed variability. Subgroup analyses did not reveal statistically significant positive effects of CT in healthy populations.

The superior cognitive benefits of CT in older adults (g = 0.33) align with the neuroprotective properties of myokine signaling (Coelho-Junior et al., 2022), which may compensate for age-related declines in cardiovascular-driven BDNF production (Erickson et al., 2011). In contrast, younger populations showed non-significant responses (p = 0.099), potentially due to their higher baseline cognitive resilience (Opdebeeck et al., 2016; Stern, 2002). The critical role of exercise order (SRT preceding AET) supports the "anabolic priming" hypothesis(Timmons et al., 2018): SRT primes the body's protein synthesis pathways, while subsequent AET takes advantage of remaining metabolic flexibility to sustain the release of neurotrophic factors. Conversely, reversed order may induce premature glycogen depletion, limiting AET's neuroplasticity effects (Fyfe et al., 2014).

### Main effects

The overall findings indicate moderate effects of CT on global cognition. This finding is consistent with previous research (Bae et al., 2019; Huang et al., 2022) which suggests that combined exercise modalities improve cognitive health, particularly in older adults (Colcombe & Kramer, 2003). The observed positive effects may be attributed to the synergistic benefits of aerobic and resistance training, which enhance neuroplasticity, increase cerebral blood flow, and promote the release of neurotrophic factors [i.e., BDNF, Insulin-like growth factor-1 (IGF-1)] and myokines (i.e., Irisin and Cathepsin B) (Cassilhas et al., 2007; Cotman & Berchtold, 2002; Kim et al., 2019; Pereira et al., 2007; Tsai et al., 2019). These cognitive improvements may be mediated by enhanced synaptic plasticity and reduced neuroinflammation, as suggested by previous studies (Voss et al., 2013). Additionally, the combination of aerobic and resistance training may offer unique benefits by targeting multiple aspects of health, such as cardiovascular fitness and muscular strength, both of which are critical for maintaining physical and cognitive function in older populations (Iso-Markku et al., 2024). In summary, the cognitive benefits of CT are driven by a complex interplay of physiological and neurological mechanisms. Future research should further investigate these mechanisms in multimodal versus single-modality exercise, with the goal of informing targeted exercise prescriptions to optimize cognitive function across the lifespan.

### Moderator analyses

Firstly, the age of participants significantly influences cognitive improvement. Adults aged 65 years and older demonstrated substantial cognitive benefits from exercise, while those aged 18 to 65 did not show significant effects (p = 0.099). This conclusion is corroborated by previous studies (Hertzog et al., 2008; Kramer & Erickson, 2007b; Paterson & Warburton, 2010), which suggest that cognitive decline associated with aging may heighten sensitivity to exercise-induced adaptations. Another possible explanation for this is the enhanced potentially for neuroplasticity and cognitive reserve in aging population (Stern, 2002). Secondly, prioritizing SRT within the exercise programs markedly promotes cognitive function, whereas prioritizing AET fail to achieve statistically significant improvements. This may be elucidated by the theory of ‘cognitive resources,’ which posits that CT modalities compete for limited neural resources, potentially resulting in cognitive fatigue (McMorris, 2020; Tomporowski & Pesce, 2019). Specifically, intra-session SRT prior to AET may be associated with stronger neuromuscular activation (Carroll et al., 2001) and a shorter recovery window (Hillman et al., 2003), while prioritizing AET, characterized by prolonged endurance exercise, may induce cognitive fatigue (Y.-K. Chang et al., 2012), weaken interaction effects (McMorris et al., 2011), and create differences in recovery periods, ultimately diminish cognitive benefits (McNerney & Radvansky, 2015). Another source of uncertainty highlighted by both our results and previous research, moreover, may interfere with each other due to neural competition between AET and SRT (Kramer & Erickson, 2007a; Lan et al., 2018). Animal studies suggest that SRT may produce negative effect on cognitive improvement induced by AET (Lan et al., 2018), which could explain the inconsistent improvements observed when AET precedes SRT (p = 0.08). Consequently, future research should investigate the optimal sequence of exercise to maximise cognitive adaptations.

Our findings indicate that short- to medium-term interventions (4–26 weeks) demonstrate positive effects on cognitive improvement. These outcomes may reflect the adaptive responses to prolonged exercise, where participants acclimate to repeated stimuli, resulting in diminished impact over time (Kraemer & Ratamess, 2005). The impacts of short- and medium-term interventions is closely related to participants’ motivation and engagement (McAuley & Rudolph, 1995), as these interventions can sustain enthusiasm, provide cognitive feedback, and enhance adherence due to appropriate length (Teixeira et al., 2012). In contrast, longer interventions may increase feelings of fatigue or reduce willingness to participate due to a lack of novelty, thereby affecting cognitive improvement outcomes (Kahn et al., 2002). These results highlight the necessity of larger sample sizes and carefully tailored interventions to capture meaningful changes in cognition. Short- to medium-term programs should therefore be prioritised to optimize cognitive benefits. Session duration results suggest that 30 to 60 minutes is optimal for enhancing cognitive function. Current research suggests that moderate-duration exercise improves neural plasticity by increasing cerebral blood flow, promoting the release of neurotrophic factors, and enhancing metabolic health (Erickson et al., 2011; Kramer & Erickson, 2007b; Liu-Ambrose et al., 2010). In contrast, 90-minute exercise sessions may lead to fatigue, reduced engagement, and diminished cognitive performance (Boksem & Tops, 2008; Ehlers et al., 2017). Longer durations do not necessarily yield additional cognitive benefits than shorter and more efficient workouts, consistent with the principle of diminishing returns (Kramer & Erickson, 2007b). Thus, interventions targeting cognitive health should prioritise exercise durations of 30 to 60 minutes for optimal benefits.

Assessment tools, including the MMSE, ADAS-Cog, and MoCA, exhibit high sensitivity to cognitive changes induced by exercise (p < 0.001). These tools are widely utilized among older adults because they assess global cognitive function across multiple domains, capturing subtle yet meaningful improvements (Folstein et al., 1975; Nasreddine et al., 2005). The MMSE and MoCA also evaluate language, visuospatial abilities, and attention, rendering them suitable for assessing the comprehensive effects of exercise on brain health. Utilizing sensitive assessment tools is crucial for detecting significant cognitive changes, particularly in complex domains such as executive function and memory (Colcombe & Kramer, 2003). Therefore, practitioners must exercise caution when selecting assessment tools for intervention studies related to cognitive health to ensure that genuine cognitive changes are accurately captured.

Finally, distinguishing between active and passive control conditions is vital for interpreting the effects of CT on cognitive health. Active controls, including health education, exercise interventions, and social activities, inherently involve cognitive engagement or physical activity, thereby influencing outcomes differently from passive controls. Structured exercise provides superior cognitive benefits compared to passive controls, indicating that it offers cognitive advantages beyond routine activities. These findings partially align with existing research (Northey et al., 2018), which indicates that exercise interventions are notably effective compared to passive control groups (e.g., wait-list), while the effects are not significant when compared to active control groups (e.g., stretching training). Thus, understanding the relationship between active and passive control conditions is crucial for optimizing the cognitive benefits of exercise-induced effects.

### Subgroup analysis and meta-regression

The substantial heterogeneity (I² = 86%) identified in our meta-analysis is noteworthy. Meta-regression and subgroup analyses revealed that variability was driven by factors such as participant age, clinical status, and exercise characteristics. Older adults and clinical populations, who are at greater risk for cognitive decline, showed the most pronounced cognitive improvements. In contrast, healthy populations exhibited non-significant trends, indicating that while exercise may have a protective effect, it does not necessarily yield significant cognitive gains in individuals without pre-existing cognitive impairments.

### Psychosocial moderators: Mental health, socioeconomics, and personality

Beyond physiological mechanisms, psychosocial factors may critically shape the cognitive outcomes of CT. First, emerging evidence suggests that CT may uniquely benefit individuals with depression or schizophrenia by addressing both cognitive deficits and psychomotor retardation. For example, SRT enhances dopaminergic signaling, which is often dysregulated in schizophrenia (Firth et al., 2017), whereas AET ameliorates hippocampal atrophy in depression (Pajonk et al., 2010). Future trials should prioritise standardised assessments of psychiatric symptoms (e.g., PHQ-9 for depression) to disentangle the bidirectional effects of exercise on mental and cognitive health (Kandola et al., 2019; Kroenke et al., 2001). While our analysis did not directly assess mental health outcomes, prior trials suggest that CT may synergistically improve both cognition and emotional well-being (Schuch, Vancampfort, Richards, et al., 2016). Second, socioeconomic determinants such as education and income likely influence intervention efficacy (Tomporowski & Pesce, 2019). Higher socioeconomic status correlates with greater access to supervised training facilities and health literacy, which may explain variability in adherence rates across studies (Stringhini et al., 2017). Third, personality traits—particularly conscientiousness and openness—may predict long-term exercise adherence, thereby indirectly moderating cognitive gains (McCrae & Sutin, 2018). Future research should explicitly measure these variables to disentangle their roles.

### Limitations and future research perspectives

The generalisability of these findings is constrained by several limitations. Firstly, significant heterogeneity exists within the sample. The 35 included RCTs featured participants spanning a broad age range (22.4 - 92.1 years), with varying health statuses and sample sizes. This population diversity may contribute to the observed heterogeneity in the results. Secondly, differences in the design and methodology—such as training configuration, intervention duration, and types of exercise—may affect the comparability and generalizability of the results. Additionally, some studies lacked detailed reporting on exercise intensity and load, limiting deeper insights into the intervention effects. Fourthly, despite subgroup analyses, the substantial heterogeneity in exercise protocols (e.g., intensity, session order, and progression) across studies may confound the interpretation of optimal CT configurations. Fifthly, our study did not systematically evaluate the role of psychosocial factors (e.g., baseline mental health, socioeconomic status, or personality traits) and psychiatric comorbidities (e.g., depression, anxiety) due to inconsistent reporting in the included trials. Finally, the absence of mechanistic evidence (e.g., neuroimaging, biomarkers) restricts our understanding of sustained cognitive benefits and underlying pathways. Notably, current research predominantly focuses on older adults, while studies investigating the cognitive effects of comprehensive exercise interventions in children and adolescents remain scarce. Thus, these results should be interpreted with caution. Future high-quality RCTs are necessary to investigate the effects of exercise interventions across age groups and to validate the long-term impacts of various exercise combinations, thereby enabling the development of more targeted exercise prescriptions. The integration of neuroimaging and biomarker analyses will enhance our understanding of how exercise influences brain structure and function, elucidating the physiological and psychological mechanisms through which exercise promotes cognitive health and advancing the development of sports medicine strategies.

### Practical application

Our findings offer actionable insights for designing cognitive health prevention programs. First, prioritizing CT with a focus on SRT before AET within a single session may maximise cognitive benefits, particularly for older adults and clinical populations at risk of neurodegeneration. Public health initiatives could integrate CT into community-based exercise programs, emphasizing supervised sessions of 30–60 minutes, 3–4 times per week, over 13–26 weeks. Second, healthcare providers may adopt CT as a non-pharmacological intervention to delay cognitive decline in individuals with MCI or early-stage dementia, given its low cost and minimal side effects. Finally, policymakers should consider promoting combined exercise regimens (e.g., combining strength and balance training with cognitive challenges) in national guidelines to enhance both physical and cognitive resilience across the lifespan.

## Conclusions

Concurrent training yield moderate improvements in cognitive health, particularly among older adults aged over 65 and clinical populations. Prioritizing resistance training, implementing short-to-medium term (4–26 weeks), and maintaining session durations of 30–60 minutes are crucial for optimizing cognitive benefits. Employing sensitive assessment tools is vital for accurately assessing cognitive changes. Further studies into optimal training protocols and multiple domains, will deepen our understanding of exercise as a form of preventive medicine for cognition. Future prevention programs should prioritise the integration of concurrent training into routine healthcare for aging populations, particularly through community centres and digital platforms that provide accessible, structured exercise protocols. By aligning exercise prescriptions with the parameters identified in this study (e.g., session duration, frequency, and exercise sequence), stakeholders can optimize cognitive health outcomes while addressing the growing burden of neurodegenerative diseases.

## Availability of data and material

All data generated or analyzed throughout this systematic review with meta-analysis were included in the main article and its supplementary materials.

## Declaration of competing interest

The authors declare the following financial interests/personal relationships which may be considered as potential competing interests: Mingyang Zhang reports article publishing charges and statistical analysis were provided by Jishou University. Wangfan Fang reports a relationship with Jishou University that includes: non-financial support. None has patent pending to None. We have nothing to declare. If there are other authors, they declare that they have no known competing financial interests or personal relationships that could have appeared to influence the work reported in this paper.

## Appendix Supplementary materials

Image, application 1
