# Supplementary material for: Effects of human concurrent aerobic and resistance training on cognitive health: A systematic review with meta-analysis
Source: Int J Clin Health Psychol. 2025 Mar 20;25(1):100559. doi: 10.1016/j.ijchp.2025.100559 (PMC11987655; doi:10.1016/j.ijchp.2025.100559)
Supplement: Supplementary file 1 [file mmc1.docx]

**Supplementary Information S1. Databases and the literature of corresponding search strategy**

| Table S1: Databases and the literature of corresponding search strategy | | |
| --- | --- | --- |
| Database | Search string | N |
| English database | | |
| PubMed | (("Humans"[MeSH Terms] AND "Cognition"[MeSH Terms] AND (("concurrent"[All Fields] OR"combined"[All Fields]) OR "simultaneous"[All Fields])) AND ("strength"[All Fields] OR "resistance"[All Fields] OR "strengthen"[All Fields])) AND ("endurance"[All Fields] OR "aerobic"[All Fields] OR "cardiovascular"[All Fields] OR " cardiorespiratory "[All Fields]) | 2870 |
| Web of Science | TS=Humans OR people AND cognition OR global cognition OR cognitive health OR neurocognitive function OR cognitive function AND concurrent training OR combined training AND strength exercise OR muscle-strengthen exercise OR resistance exercise OR resistance training OR strength training AND aerobic training OR aerobic exercise OR endurance training OR endurance exercise OR cardiorespiratory training OR cardiorespiratory exercise OR cardiovascular training OR cardiovascular exercise OR physical exercise OR exercise training OR physical activity | 2430 |
| EMBASE | TS=Humans OR people AND cognition OR global cognition OR cognitive health OR neurocognitive function OR cognitive function AND concurrent training OR combined training AND strength exercise OR muscle-strengthen exercise OR resistance exercise OR resistance training OR strength training AND aerobic training OR aerobic exercise OR endurance training OR endurance exercise OR cardiorespiratory training OR cardiorespiratory exercise OR cardiovascular training OR cardiovascular exercise OR physical exercise OR exercise training OR physical activity | 2120 |
| CINAHL | TS=Humans OR people AND cognition OR global cognition OR cognitive health OR neurocognitive function OR cognitive function AND concurrent training OR combined training AND strength exercise OR muscle-strengthen exercise OR resistance exercise OR resistance training OR strength training AND aerobic training OR aerobic exercise OR endurance training OR endurance exercise OR cardiorespiratory training OR cardiorespiratory exercise OR cardiovascular training OR cardiovascular exercise OR physical exercise OR exercise training OR physical activity | 1450 |
| Scopus | TS=Humans OR people AND cognition OR global cognition OR cognitive health OR neurocognitive function OR cognitive function AND concurrent training OR combined training AND strength exercise OR muscle-strengthen exercise OR resistance exercise OR resistance training OR strength training AND aerobic training OR aerobic exercise OR endurance training OR endurance exercise OR cardiorespiratory training OR cardiorespiratory exercise OR cardiovascular training OR cardiovascular exercise OR physical exercise OR exercise training OR physical activity | 480 |
| Google Scholar | TS=Humans OR people AND cognition OR global cognition OR cognitive health OR neurocognitive function OR cognitive function AND concurrent training OR combined training AND strength exercise OR muscle-strengthen exercise OR resistance exercise OR resistance training OR strength training AND aerobic training OR aerobic exercise OR endurance training OR endurance exercise OR cardiorespiratory training OR cardiorespiratory exercise OR cardiovascular training OR cardiovascular exercise OR physical exercise OR exercise training OR physical activity | 3200 |
| Cochrane Library | TS=Humans OR people AND cognition OR global cognition OR cognitive health OR neurocognitive function OR cognitive function AND concurrent training OR combined training AND strength exercise OR muscle-strengthen exercise OR resistance exercise OR resistance training OR strength training AND aerobic training OR aerobic exercise OR endurance training OR endurance exercise OR cardiorespiratory training OR cardiorespiratory exercise OR cardiovascular training OR cardiovascular exercise OR physical exercise OR exercise training OR physical activity | 310 |
| Chinese database | | |
| CNKI | 1. TS=Humans OR people AND cognition OR global cognition OR cognitive health OR neurocognitive function OR cognitive function AND concurrent training OR combined training AND strength exercise OR muscle-strengthen exercise OR resistance exercise OR resistance training OR strength training AND aerobic training OR aerobic exercise OR endurance training OR endurance exercise OR cardiorespiratory training OR cardiorespiratory exercise OR cardiovascular training OR cardiovascular exercise OR physical exercise OR exercise training OR physical activity 2. （同期训练 or 同期运动 or 同期有氧和力量训练 or 同期有氧和抗阻训练 or 有氧与抗阻联合运动）AND（认知 or 认知功能 or 认知表现 or 认知健康 or 认知能力）AND (随机对照试验 or RCT or randomized controlled trail or 随机对照实验) | 369 |
| Baidu Scholar | ① TS=Humans OR people AND cognition OR global cognition OR cognitive health OR neurocognitive function OR cognitive function AND concurrent training OR combined training AND strength exercise OR muscle-strengthen exercise OR resistance exercise OR resistance training OR strength training AND aerobic training OR aerobic exercise OR endurance training OR endurance exercise OR cardiorespiratory training OR cardiorespiratory exercise OR cardiovascular training OR cardiovascular exercise OR physical exercise OR exercise training OR physical activity  ②（同期训练 or 同期运动 or 同期有氧和力量训练 or 同期有氧和抗阻训练 or 有氧与抗阻联合运动）AND（认知 or 认知功能 or 认知表现 or 认知健康 or 认知能力）AND (随机对照试验 or RCT or randomized controlled trail or 随机对照实验) | 240 |
| WanFang Data | 1. TS=Humans OR people AND cognition OR global cognition OR cognitive health OR neurocognitive function OR cognitive function AND concurrent training OR combined training AND strength exercise OR muscle-strengthen exercise OR resistance exercise OR resistance training OR strength training AND aerobic training OR aerobic exercise OR endurance training OR endurance exercise OR cardiorespiratory training OR cardiorespiratory exercise OR cardiovascular training OR cardiovascular exercise OR physical exercise OR exercise training OR physical activit 2. （同期训练 or 同期运动 or 同期有氧和力量训练 or 同期有氧和抗阻训练 or 有氧与抗阻联合运动）AND（认知 or 认知功能 or 认知表现 or 认知健康 or 认知能力）AND (随机对照试验 or RCT or randomized controlled trail or 随机对照实验) | 175 |
| Following literature review | | |
| Consemnsus | AI search: “concurrent aerobic and resistance training” / “combined aerobic and resistance exercise” AND “cognition” | 52 |
| Elicit | AI search: “concurrent aerobic and resistance training” / “combined aerobic and resistance exercise” AND “cognition” | 76 |
| Core reference lists | Bsed on umbrella review, literature review, systematic review with meta analysis | 62 |

**Supplementary Information S2.****The classifications of** **study design and concurrent training configuration**

Table S2. The classifications of study design and concurrent training configuration

| **Age** | |
| --- | --- |
| Child and adolescence | 6-18 years |
| Young adulthood | 18-40 years |
| Middle adulthood | 40-65 years |
| Old adults | ＞65 years |
| **Health status** | |
| Healthy population | Human without diseases and cognitively impaired |
| Clinical population | clinical physiological conditions (e.g., diabetes, breast cancer, frailty) and neurocognitive disorders/decline [e.g., mild cognitive impairment (MCI), dementia, Alzheimer’s disease (AD), and multiple sclerosis (MS)] |
| **Frequency** | |
| Low | 1-2 times/week |
| moderate | 3-4 times/week |
| High | ≥5 times/week |
| **Intensity** | |
| Sedentary | Aerobic exercise: <20% V̇O_2_max，<40% HRmax Resistance exercise: RPE <8 |
| Low | Aerobic exercise:  20-40% V̇O_2_max/HRR, 40-55% HRmax  Resistance exercise: < 50% 1-RM |
| Moderate | Aerobic exercise: 40-60% V̇O_2_max/HRR, 55%-70% HR max  Resistance exercise: 50–70% 1-RM |
| High | Aerobic exercise: 61-85% V̇O_2_max/HRR, 71-90% HR max  Resistance exercise: 71-84% 1-RM |
| Load Progression | ↑Change in exercise intensity throughout intervention |
| **Overall length** | |
| Short | 4-12 weeks (1-3 months) |
| moderate | 13-26 weeks (3-6 months) |
| Long | >26 weeks (>6 months) |
| **Type** | |
| Aerobic exercise | aiming to improve cardiovascular fitness including walking, running, swimming, rowing, cycling, dance-based aerobics, or jumping jacks |
| Resistance exercise | with the intent of increasing muscular strength and power using body-weight, free weights, weight-lifting machines, or resistance bands |
| **Duration of exercise bout** | |
| Short | 30-45 min |
| moderate | 60 min |
| Long | ＞60 min |

**Supplementary Information S3.1 Quality Assessment Across Studies: A Systematic Overview**


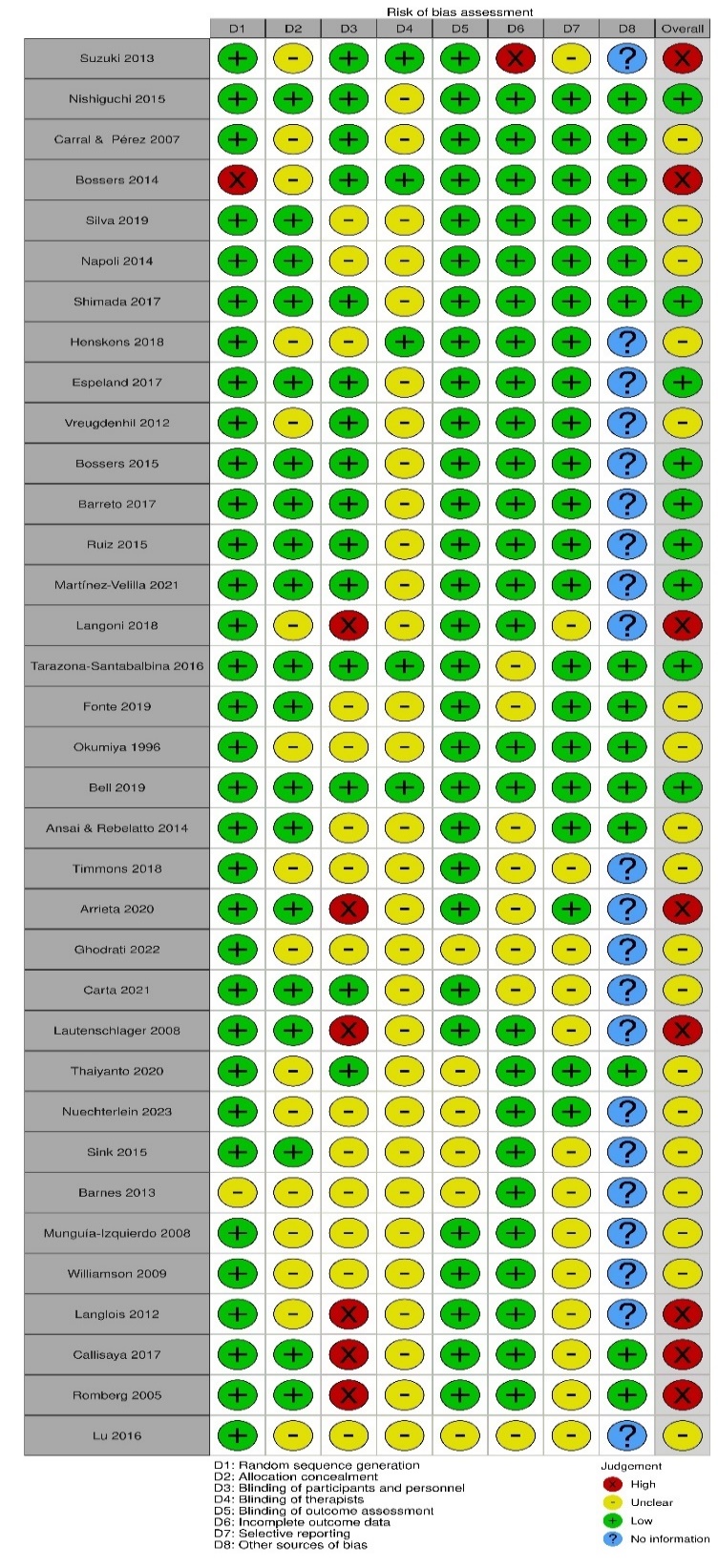


Figure S3. Quality Assessment Across Studies: A Systematic Overview

**Supplementary Information S3.2** **Methodological quality assessment of included studies**

| Table S3 Methodological quality assessment of included studies | | | | | | | | | | |
| --- | --- | --- | --- | --- | --- | --- | --- | --- | --- | --- |
| Study | Random.sequence.generation | Allocation.concealment | Blinding.of.participants.and.personnel | | Blinding.of.therapists | Blinding.of.outcome.assessment | Incomplete.outcome.data | Selective.reporting. | Other.sources.of.bias. | Overall |
| Suzuki 2013 | Low | Unclear | | High | Low | Low | High | Unclear | No information | High |
| Nishiguchi 2015 | Low | Low | | High | Unclear | Low | Low | Low | Low | Low |
| Carral & Pérez 2007 | Low | Unclear | | High | Unclear | Low | Low | Low | Low | Unclear |
| Bossers 2014 | High | Unclear | | High | Low | Low | Low | Low | Low | High |
| Silva 2019 | Low | Low | | High | Unclear | Low | Low | Low | Low | Unclear |
| Napoli 2014 | Low | Low | | High | Unclear | Low | Low | Low | Low | Unclear |
| Shimada 2017 | Low | Low | | High | Unclear | Low | Low | Low | Low | Low |
| Henskens 2018 | Low | Unclear | | High | Low | Low | Low | Low | No information | Unclear |
| Espeland 2017 | Low | Low | | High | Unclear | Low | Low | Low | No information | Low |
| Vreugdenhil 2012 | Low | Unclear | | High | Unclear | Low | Low | Low | No information | Unclear |
| Bossers 2015 | Low | Low | | High | Unclear | Low | Low | Low | No information | Low |
| Barreto 2017 | Low | Low | | High | Unclear | Low | Low | Low | No information | Low |
| Ruiz 2015 | Low | Low | | High | Unclear | Low | Low | Low | No information | Low |
| Martínez-Velilla 2021 | Low | Low | | High | Unclear | Low | Low | Low | No information | Low |
| Langoni 2018 | Low | Unclear | | High | Unclear | Low | Low | Unclear | No information | High |
| Tarazona-Santabalbina 2016 | Low | Low | | High | Low | Low | Unclear | Low | Low | Low |
| Fonte 2019 | Low | Low | | High | Unclear | Low | Unclear | Low | Low | Unclear |
| Okumiya 1996 | Low | Unclear | | High | Unclear | Low | Low | Low | Low | Unclear |
| Bell 2019 | Low | Low | | High | Low | Low | Low | Low | Low | Low |
| Ansai & Rebelatto 2014 | Low | Low | | High | Unclear | Low | Unclear | Low | Low | Unclear |
| Timmons 2018 | Low | Unclear | | High | Unclear | Low | Unclear | Unclear | No information | Unclear |
| Arrieta 2020 | Low | Low | | High | Unclear | Low | Unclear | Low | No information | High |
| Ghodrati 2022 | Low | Unclear | | High | Unclear | Unclear | Unclear | Unclear | No information | Unclear |
| Carta 2021 | Low | Low | | High | Unclear | Low | Unclear | Unclear | No information | Unclear |
| Lautenschlager 2008 | Low | Low | | High | Unclear | Low | Low | Unclear | No information | High |
| Thaiyanto 2020 | Low | Unclear | | High | Unclear | Unclear | Low | Low | Low | Unclear |
| Nuechterlein 2023 | Low | Unclear | | High | Unclear | Unclear | Low | Low | No information | Unclear |
| Sink 2015 | Low | Low | | High | Unclear | Unclear | Low | Unclear | No information | Unclear |
| Barnes 2013 | Unclear | Unclear | | High | Unclear | Unclear | Low | Unclear | No information | Unclear |
| Munguía-Izquierdo 2008 | Low | Unclear | | High | Unclear | Low | Low | Unclear | No information | Unclear |
| Williamson 2009 | Low | Unclear | | High | Unclear | Low | Low | Unclear | No information | Unclear |
| Langlois 2012 | Low | Unclear | | High | Unclear | Low | Low | Unclear | No information | High |
| Callisaya 2017 | Low | Low | | High | Unclear | Low | Low | Unclear | Low | High |
| Romberg 2005 | Low | Low | | High | Unclear | Low | Low | Unclear | Low | High |
| Lu 2016 | Low | Unclear | | High | Unclear | Unclear | Unclear | Unclear | No information | Unclear |
|  | | | | | | | | | | |

**Supplementary Information S4. Statistical significance and right-skewness in empirical p-curve analysis**


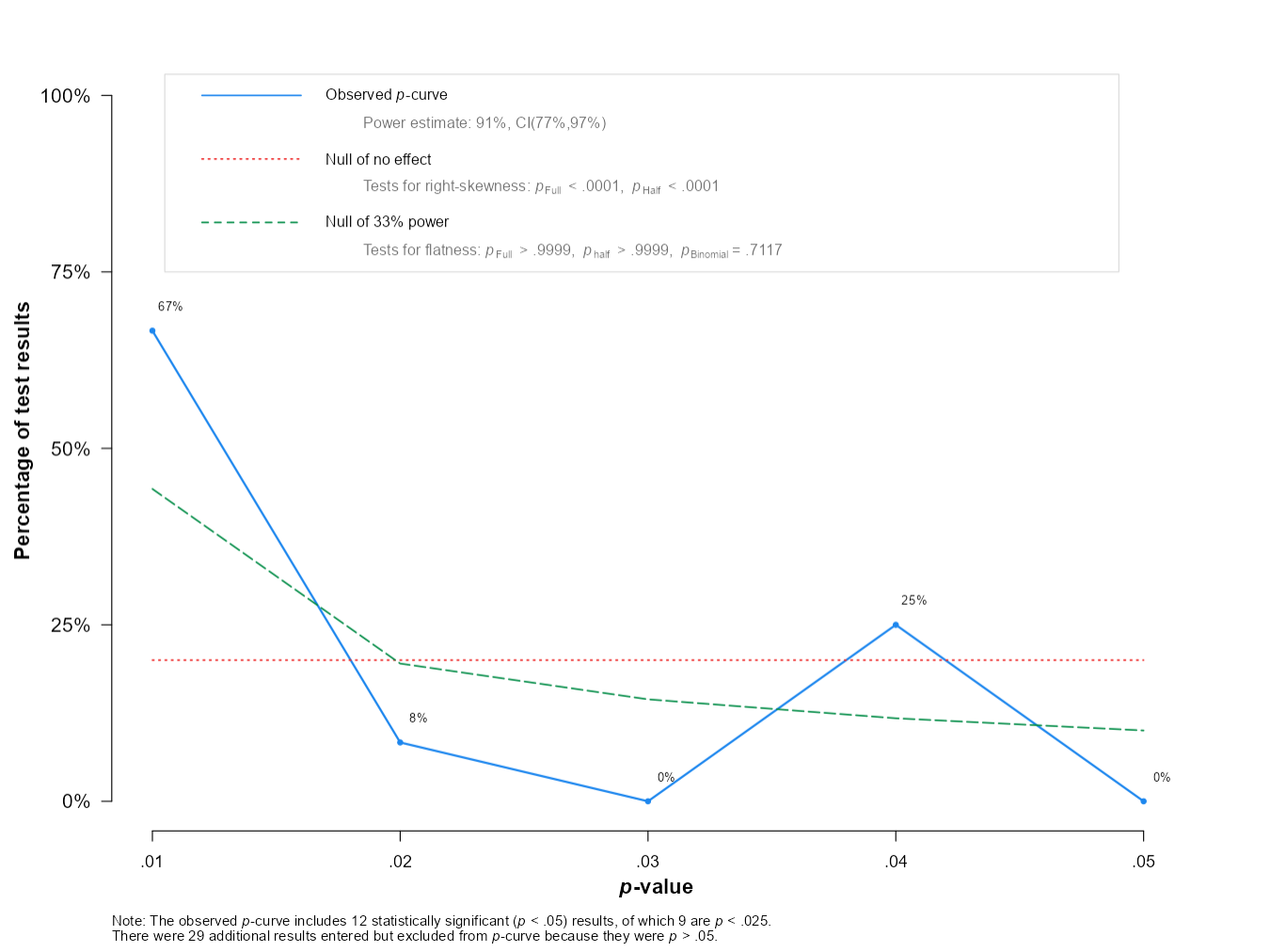


Figure S4. Statistical significance and right-skewness in empirical p-curve analysis

**Supplementary Information S5.** **Funnel plot of included dependent effect sizes.**


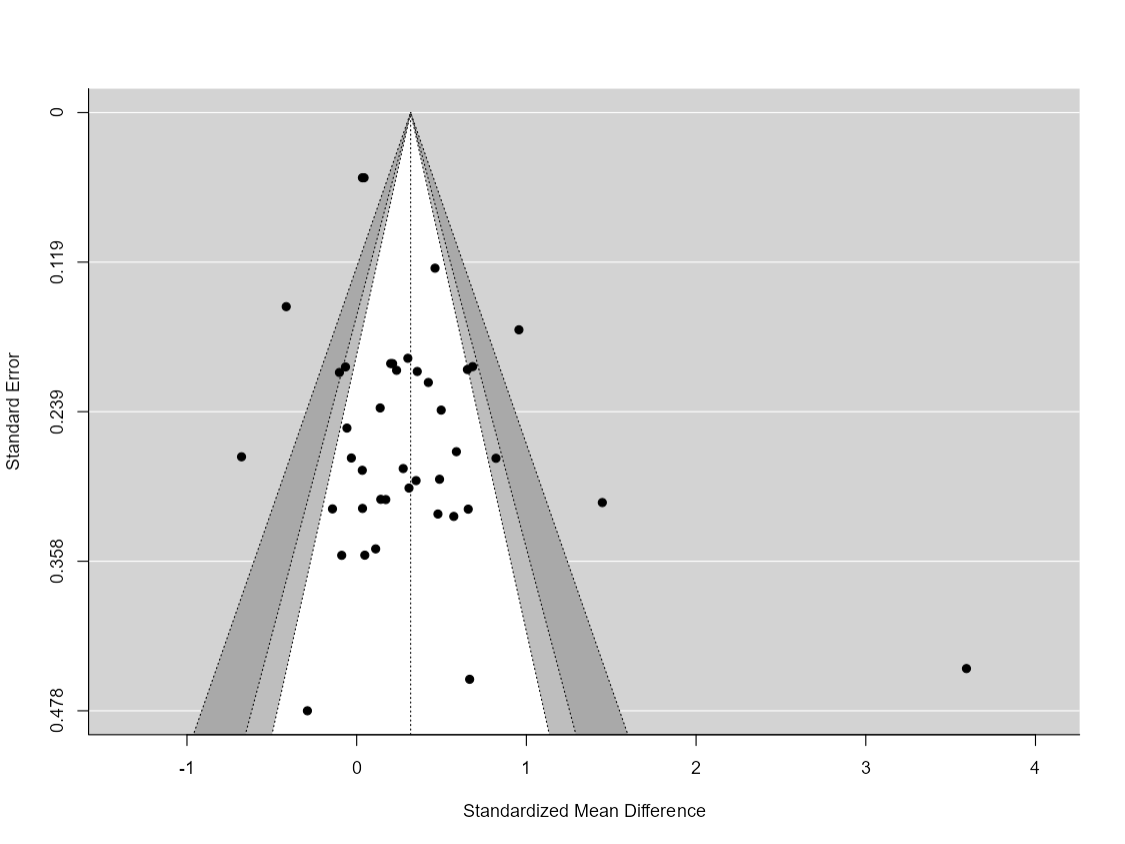


Figure S5. Funnel plot of risk of bias assessment

**Supplementary Information S6. Forest plot of moderator analysis: impact of health status on cognitive outcomes**


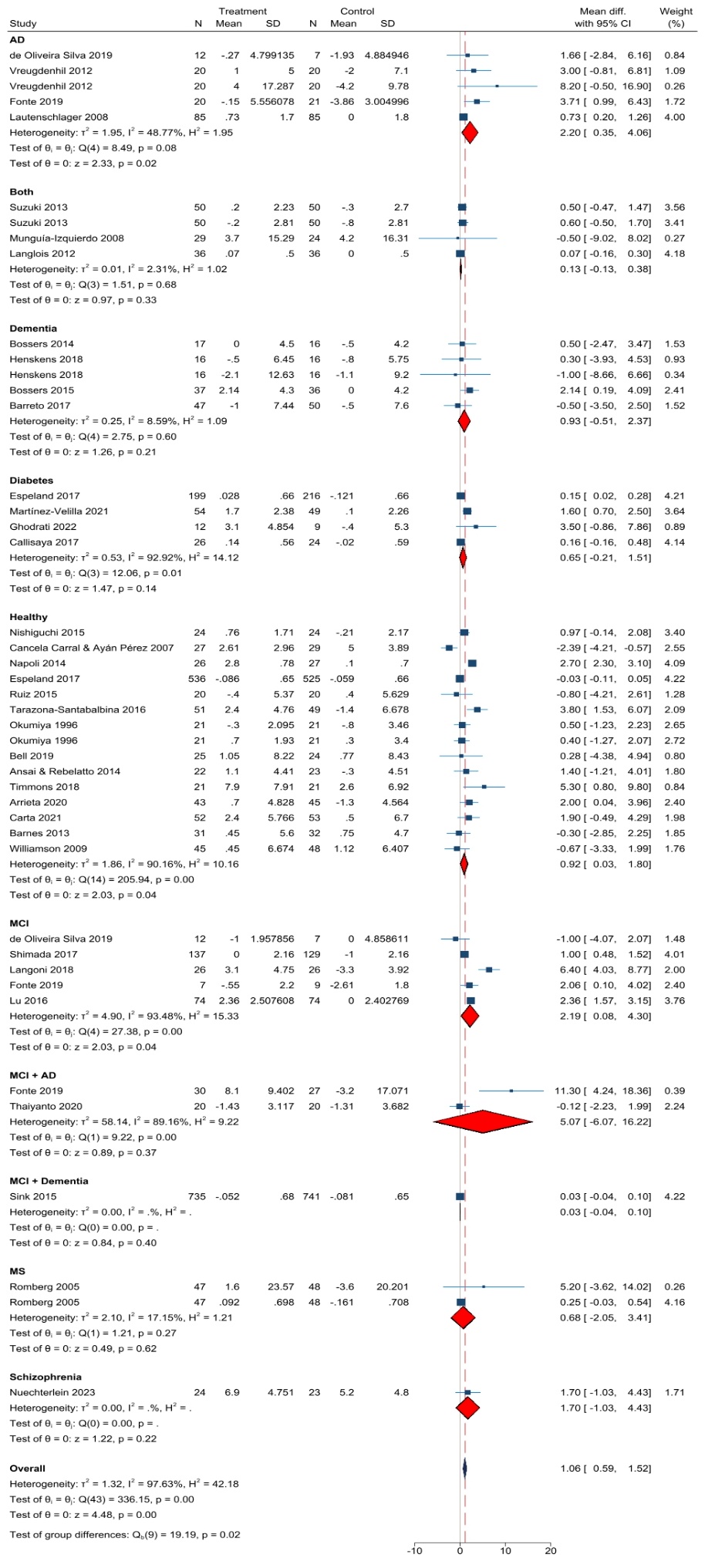


Figure S6. Forest plot of moderator analysis: impact of participants’ health status on cognitive outcomes. CI, confidence interval; IV, inverse variance; SD, standard deviation.

**Supplementary Information S7. Forest plot of overall factor analyses on cognitive outcomes**


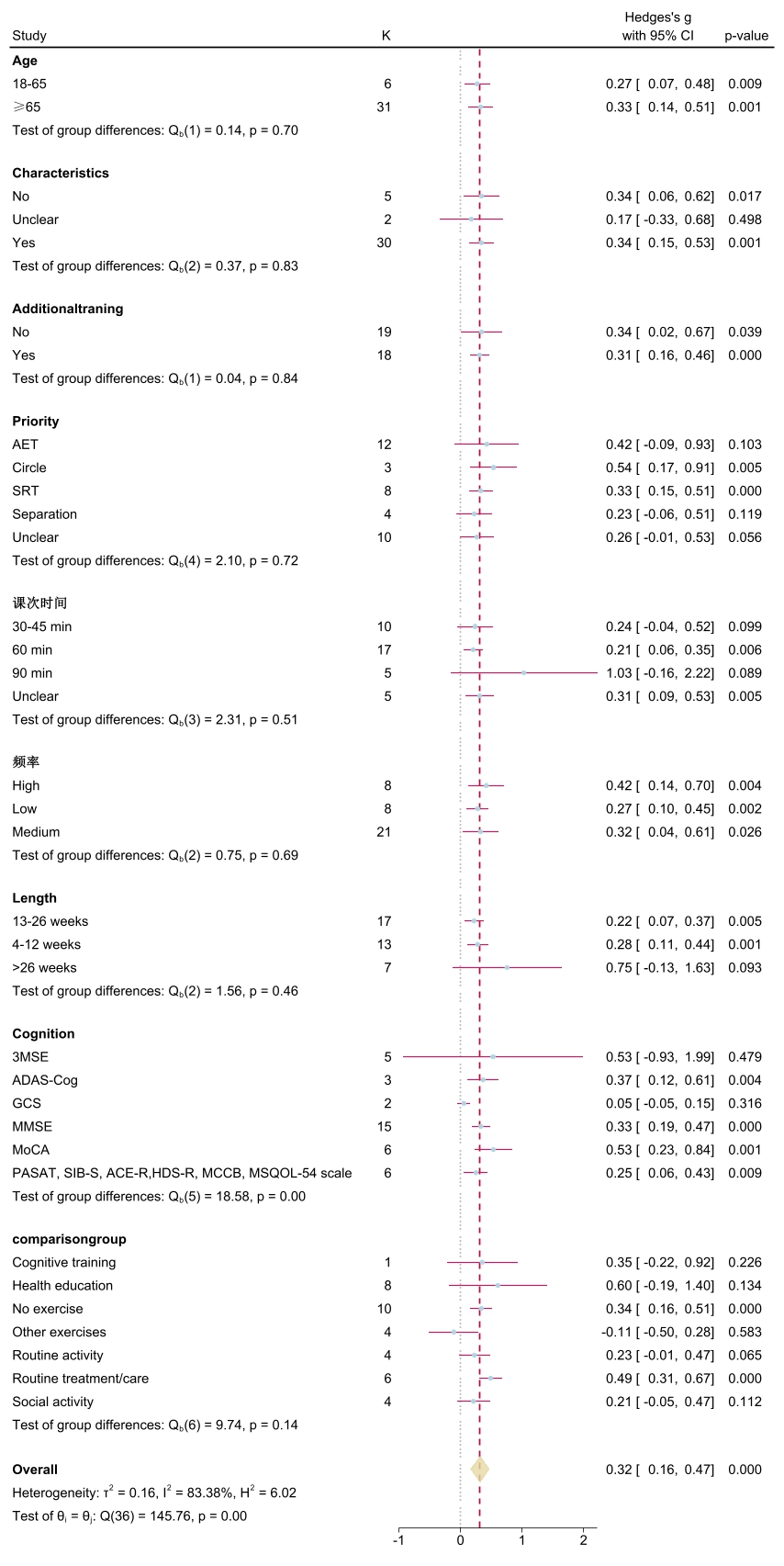


Figure 11. Forest plot of overall factor analyses. K, number of effect sizes; CI, confidence interval.
